# A systems biological analysis of the ATF4‐GADD34‐CHOP regulatory triangle upon endoplasmic reticulum stress

**DOI:** 10.1002/2211-5463.13484

**Published:** 2022-09-27

**Authors:** Margita Márton, Gábor Bánhegyi, Norbert Gyöngyösi, Eszter Éva Kálmán, Aladár Pettkó‐Szandtner, Krisztina Káldi, Orsolya Kapuy

**Affiliations:** ^1^ Department of Molecular Biology at the Institute of Biochemistry and Molecular Biology Semmelweis University Budapest Hungary; ^2^ Laboratory of Proteomics Research Biological Research Centre Szeged Hungary; ^3^ Department of Physiology Semmelweis University Budapest Hungary

**Keywords:** ATF4‐GADD34‐CHOP, endoplasmic reticulum stress, feedback loop, PERK pathway, systems biology, unfolded protein response

## Abstract

Endoplasmic reticulum (ER) stress‐dependent accumulation of incorrectly folded proteins leads to activation of the unfolded protein response. The role of the unfolded protein response (UPR) is to avoid cell damage and restore the homeostatic state by autophagy; however, excessive ER stress results in apoptosis. Here we investigated the ER stress‐dependent feedback loops inside one of the UPR branches by focusing on PERK‐induced ATF4 and its two targets, called CHOP and GADD34. Our goal was to qualitatively describe the dynamic behavior of the system by exploring the key regulatory motifs using both molecular and theoretical biological techniques. Using the HEK293T cell line as a model system, we confirmed that the life‐or‐death decision is strictly regulated. We investigated the dynamic characteristics of the crucial elements of the PERK pathway at both the RNA and protein level upon tolerable and excessive levels of ER stress. Of particular note, inhibition of GADD34 or CHOP resulted in various phenotypes upon high levels of ER stress. Our computer simulations suggest the existence of two new feedback loops inside the UPR. First, GADD34 seems to have a positive effect on ATF4 activity, while CHOP inhibits it. We claim that these newly described feedback loops ensure the fine‐tuning of the ATF4‐dependent stress response mechanism of the cell.

## Graphical abstract

Key components of the endoplasmic reticulum stress‐activated PERK pathway are ATF4, GADD34, and CHOP. These molecules play important roles in the cellular decision between autophagy and apoptosis. Using systems biological tools, we suggest new relationships (blue lines) that create feedback loops inside the PERK pathway of the unfolded protein response.

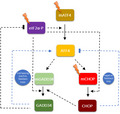

**Abbreviations**
ER: endoplasmic reticulum
GB: guanabenz
TG: thapsigargin
UPR: unfolded protein response

For adequate function of the cell, it is essential to preserve cellular homeostasis against various physiological and pathological changes. Endoplasmic reticulum (ER) is a complex eukaryotic organelle where both membrane and secretory proteins are assembled into their final proper conformation. Besides, ER works as a molecular gateway for the secretory pathway [1, 2]. Exaggerated protein secretion or various defects in the protein folding process may lead to the accumulation of misfolded proteins, which alters ER homeostasis and results in ER stress [3, 4].

Cellular self‐cannibalism, called autophagy, is one of the most important mechanisms of the cell maintaining its homeostasis in case of various ER stress [5, 6]. Autophagy is an evolutionally conserved, well‐controlled process where unfolded protein aggregates and damaged organelles are sequestered in a double‐membrane vesicle, called the autophagosome. After the fusion of autophagosome with lysosome, autolysosome gets formed to guarantee the quick, acidic digestion of these toxic cytosolic components. Therefore, autophagy eliminates cellular stress factors for promoting cell survival upon ER stress [7, 8]. However, unresolved ER stress generates apoptotic cell death [9]. Apoptosis is a genetically programmed cell‐death process, whereby the organism removes unnecessary or malfunctioning cells in an ATP‐dependent way [10]. Apoptotic cells are well‐characterized by cell shrinkage, the loss of connections with other cells, chromatin condensation, and fragmentation by endonucleases, among others. At the end of this process, cell breaks up to small double‐membrane vesicle pieces called apoptotic bodies, which are eliminated quickly by macrophages, thereby avoiding cellular inflammation [9, 11].

It has been already shown that ER stress can activate both autophagy and apoptosis [12]. By our previous findings [13], activation of these two processes requires a defined order upon ER stress. Even under mild, so‐called “low‐level ER stress,” autophagy is always activated to restore cell homeostasis. However, in case of longer or nontolerable ER stress, a “high ER stress level” was determined where autophagy had a transient activation peak first, but later apoptosis turned on [13]. According to our previous results, the order is well‐determined between the two stress response mechanisms: autophagy always precedes apoptotic cell death due to the mutual exclusion generated by a double‐negative feedback loop between the key regulators of the two processes [13, 14, 15].

An evolutionarily conserved system of signal transduction pathways, called unfolded protein response (UPR), is always activated with respect to ER stress [16]. The main role of UPR is to guarantee the accuracy of protein folding, to maintain ER functions, to reduce the harmful effects of ER stress, and to restore ER homeostasis through autophagy induction. On the other hand, if the recovery is not possible, it promotes apoptotic cell death [3, 8]. UPR is regulated by three well‐known ER transmembrane proteins, called PERK (Protein Kinase R‐like ER kinase), ATF6 (Activating transcription factor 6) and IRE‐1 (inositol‐requiring enzyme 1) [17]. Under physiological conditions, the chaperone protein BiP (Binding immunoglobulin Protein) binds to these ER stress sensor proteins (PERK, ATF6, and IRE1), keeping them in an inactive state [18]. However, in case of ER stress conditions, BiP gets quickly released, enabling the activation of these sensor proteins [18, 19].

It has been recently introduced by our lab [14] that there is a well‐defined crosstalk between PERK and IRE1 arms of the UPR. We have shown that not only IRE1 has a positive effect on PERK [20], but PERK also enhances IRE1 activity generating a positive feedback loop between IRE1 and PERK. We showed that this positive connection is responsible for the robust, one‐way directionality of the proper response upon ER stress. In addition, we confirmed that both IRE1 and PERK could induce autophagy or apoptosis via their downstream targets, but they differed in the strength of autophagy and apoptosis induction. Therefore, through the positive feedback loop between IRE1 and PERK and the double negative feedback loop between autophagy and apoptosis inducers, the control system is able to guarantee the transient activation peak of autophagy with respect to an excessive level of ER stress; meanwhile, apoptosis has an irreversible switch‐like characteristic [14].

With respect to ER stress, it is well known that active PERK phosphorylates translation initiation factor eIF2α on Ser51, reducing global protein synthesis. This limits the entry of newly formed proteins into the ER to reduce stress, while phosphorylated eIF2α facilitates translation of those mRNAs that contain one or more uORF (upstream Open Reading Frame) in their 5′UTR [19, 21]. Therefore, phosphorylated eIF2α promotes the translation of the ATF4 transcription factor that activates expression of its key targets, called GADD34 and CHOP, respectively [22, 23]. GADD34 (growth arrest and DNA damage [GADD]‐inducible34) is a regulatory subunit of the phosphatase holoenzyme GADD34:PP1 that dephosphorylates eIF2α‐P, alleviating the translational inhibition to aid translational recovery and preserve energy sources of the cell [24, 25]. CHOP (C/EBP homologous protein) is a cellular stress sensor molecule. It is highly expressed during stress conditions and translocates to the nucleus, where it regulates transcription of several genes by binding to their promoter's CAAT‐box motif [26]. CHOP can transcriptionally activate many genes related to intrinsic and extrinsic apoptotic pathways, or the ERO1α gene which induces ROS production [26]. Although CHOP‐deleted cells are much less sensitive to ER stress compared to wildtype strain [27], it has been shown that CHOP is able to promote both autophagy‐dependent survival and apoptotic cell death upon ER stress [28]. The important cytoprotective role of GADD34 induction is also proved [29]. Interestingly both GADD34 overexpression and catalytically inactive GADD34 addition (GADD3434ΔC/ΔC) result in premature cell death in the presence of ER stress [30, 31], but the detailed mechanism is still a mystery. It has been also shown that CHOP has some positive effect on GADD34 [32, 33].

NRF2 is another transcription factor of the PERK pathway, which is directly activated by phosphorylated PERK, during stress conditions [34]. Activated NRF2 translocates to the nucleus, where it promotes transcription of many genes, mainly related to antioxidant pathways [8, 35]. Since NRF2 seems to be more important in the antioxidant response, our study does not contain or explore this component of the PERK pathway.

Since all three arms of UPR are so complex, with so many components and crosstalks [36, 37], the question immediately arises what are the key regulatory connections (i.e. positive and negative feedback loops) inside the given UPR branch. Theoretical analysis might help us to investigate the dynamical characteristic of UPR. Tang *et al*. investigated the importance of the translation attenuation mechanism in higher eukaryotes upon ER stress, but they did not take into account how the components of the PERK branch can influence each other [38]. Recently, another detailed model of UPR was developed, but the important role of ER stress response mechanism induced autophagy has not been introduced [39]. Curtu *et al*. created a nice analysis of the ER stress response mechanism in vertebrates by containing both theoretical and molecular biological techniques. However, these models did not introduce all the components of the PERK pathway, such as ATF4, CHOP, and GADD34 [40].

Our goal was to investigate whether there is any crosstalk between the key molecules inside the most well‐known UPR branch, called PERK. Since GADD34ΔC/ΔC cells can induce neither ATF4 nor CHOP activations [31], we were interested in whether not only CHOP has a positive effect on GADD34, but that GADD34 might promote CHOP activation directly or indirectly through ATF4. By using both molecular biological techniques and theoretical biological analysis, we claim that extra feedback loops inside the PERK branch guarantee the proper dynamical behavior of cellular life‐and‐death decisions upon ER stress.

## Materials and methods

### Materials

Thapsigargin (T9033; Sigma‐Aldrich, St. Louis, MO, USA) and guanabenz (G110; Sigma‐Aldrich) were purchased. All other chemicals were of reagent grade.

### Cell culture and maintenance

As a model system, human embryonic kidney (HEK293T, ATCC, CRL‐3216, Manassas, VA, USA) cell lines were used, maintained in DMEM (41965039; Life Technologies, Carlsbad, CA, USA) medium supplemented with 10% fetal bovine serum (10500064; Life Technologies) and 1% antibiotics/antimycotics (15240062; Life Technologies). Culture dishes and cell treatment plates were kept in a humidified incubator at 37 °C in 95% air and 5% CO_2_.

### SDS/PAGE and western blot analysis

Cells were harvested and lysed with 20 mm Tris, 135 mm NaCl, 10% glycerol, 1% NP40, pH 6.8. The Protein content of cell lysates was measured using the Pierce BCA Protein Assay (23225; Thermo Scientific). During each procedure, equal amounts of protein were used. SDS/PAGE was done using the SE250 Mighty Small II Mini Vertical Protein Electrophoresis Unit (Hoefer: Holliston, MA, USA). Proteins were transferred onto Millipore (Billerica, MA, USA) 0.45 μm PVDF membrane, using TE22 Mighty Small Transfer Tank (Hoefer). Immunoblotting was performed using TBS Tween (0.1%), containing 5% nonfat dry milk for blocking membrane and for antibody solutions. Loading was controlled by developing membranes for GAPDH in each experiment. The following antibodies were applied: anti‐LC3β (sc‐16755; SantaCruz: Dallas, TX, USA), anti‐PARP (9542S; Cell Signaling: Danvers, MA, USA), anti‐CHOP (15204‐1‐AP; Proteintech: Mancherster, UK), anti‐ATF4 (10835‐1‐AP; Proteintech), anti‐GADD34 (10449‐1‐AP; Proteintech), anti‐eIF2α (9722S9; Cell Signaling), anti‐P‐eIF2α (9721L; Cell Signaling), and anti‐GAPDH (6C5; Santa Cruz), anti‐TRIB3 (13300‐1‐AP; Proteintech), anti‐DNAJC3 (26721‐1‐AP; Proteintech), Anti‐Grp78 (76‐E6; Santa Cruz), and HRP conjugated secondary antibodies (7074S and 7076S; Cell Signaling). In case of detecting CHOP, the PVDF membrane was treated with glutaraldehyde solution (G5882; Sigma‐Aldrich), at a final concentration of 0.15% glutaraldehyde, for fixing the small and very acidic CHOP protein on the membrane, thus preventing its release from the membrane by the incubation steps with milk solution [41].

### RNA interference

RNA interference experiments were performed using Lipofectamine™ RNAiMAX Transfection Reagent (13778075; Invitrogen™: Waltham, MA, USA), Opti‐MEM™ Reduced Serum Medium, GlutaMAX™ Supplement (51985034; GIBCO™, Life Technologies), and DDIT3‐Silencer Pre‐Designed siRNA (AM16708; Ambion, Life Technologies) at a concentration of 20 pmol·mL^−1^. A total of 400 000 HEK293T cells were incubated at 37 °C in a CO_2_ incubator in antibiotic‐free medium for 24 h, on 6‐well plates, then the RNAi duplex‐Lipofectamine™ RNAiMAX complexes were added to the cells for 24 h. Next, fresh medium was added to the cells and the appropriate treatment was carried out.

### Separation and isolation of nuclear and cytoplasmic protein extracts

The NE‐PER nuclear and cytoplasmic extraction reagents (78833; Thermo Scientific) were used to separate and isolate cytoplasmic and nuclear proteins. In order to obtain the correct protein concentration, extraction was performed from 8.5 × 10^6^ HEK293T cells per sample. Isolation was performed according to the manufacturer's instructions. Anti‐Histone H3 (against the nuclear loading control protein, 17168‐1‐AP; Proteintech) and anti‐GAPDH (against the cytoplasmic loading control protein, Santa Cruz, 6C5) antibodies were used to check the efficiency of the separation of two fractions.

### RNA isolation, reverse transcription, and real‐time PCR

The total RNA content of cells was extracted using TRIzol RNA isolation reagent (Invitrogen) [42]. Retrotranscription was performed using the SuperScriptII First‐Strand Synthesis System (Invitrogen). Nucleic acid levels were measured using the NanoDrop2000 UV calculator. Equal amounts of cDNA were used for real‐time PCR to follow gene expressions. PCR reaction and real‐time detection was performed using PowerUp SYBR Green Master Mix (A25742; Thermo Fisher Scientific: Austin, TX, USA) and QuantStudio 12K Flex System (Thermo Fisher Scientific). Real‐time PCR thermocycles were the followings: 95 °C 10 min (1×), 95 °C 15 s, 60 °C 1 min (40x×), 95 °C 15 s, 60 °C 1 min, 97 °C 15 s (1×). The primers were as follows: for GADD34: (forward) 5′‐GACCTGTGATCGCTTCTGG‐3′ and (reverse) 5′‐TAGCCTGATGGGGTGCTT‐3′, for CHOP: (forward) 5′‐TAGCCTGATGGGGTGCTT‐3′ and (reverse) 5′‐TGGATCAGTCTGGAAAAGCA‐3′, for ATF4: (forward) 5′‐CTCATGGGTTCTCCAGCGAC‐3′ and (reverse) 5′‐GGGCATCCAAGTCGAACTCC‐3′, for GAPDH: (forward) 5′‐TGCACCACCAACTGCTTAGC‐3′ and (reverse) 5′‐GGCATGGACTGTGGTCATGAG‐3′.

### Cell viability assays

The relative amount of viable cells was calculated by Burker chambers. Cell viability was detected using CellTiter‐Blue assay (G8080; Promega, Madison, WI, USA). Cells were grown and treated on 96‐well plates and were incubated with resazurin for 2 h at 37 °C. Absorbance was measured at 620 nm, and expressed in arbitrary units, being proportional to cell toxicity. For each of these experiments at least three parallel measurements were carried out. Nontreated controls contained the same percentage of DMSO as the treated ones.

### Apo‐ONE ^®^ homogeneous Caspase‐3/7 assays

The activity of caspase‐3 and caspase‐7 were determined by using the fluorescent apoptosis assay Apo‐ONE^®^ Homogeneous Caspase‐3/7 Assay (G7790; Promega). HEK293T cells were seeded on black 96‐well clear‐bottom microplates and were grown for 24 h. After the appropriate treatment, equal volume of the Apo‐ONE^®^ reagent was added to an equal volume of cells and culture medium‐containing wells. After shaking at 300 r.p.m. for 30 s, plates were incubated at room temperature for 3 h, and the fluorescence of each well was measured at the excitation of 487 nm and emission of 535 nm by the CLARIOstar^®^ Plus Microplate Reader, using the enhanced dynamic range tool. The assay is based on the cleavage of the Caspase Substrate Z‐DEVD‐R110 by Caspase‐3/7, thereby forming the fluorescent Rhodamine 110. The fluorescence intensity of samples is proportional to the caspase‐3/7 cleavage activity. After the subtraction of fluorescence values of blank samples, the average of fluorescence intensity of the triplicates was divided by the nontreated control (containing the same percentage of DMSO as the treated ones), for the illustration of relative caspase 3/7 activity.

### TurboID experiment

The DNA construct encoding the C‐terminal chimera protein GADD34‐TurboID was cloned into pcDNA 3.1 (−) vector, and HEK293T cells were transfected by the sequenced plasmid with lipofectamin 3000 (L300‐015; Invitrogen) according to the manufacturer's instructions. After transfection, the cells were incubated in the presence or absence of 10 μm Thapsigargin for 2 h. Fifty micromolar final concentration of biotin was used for *in vivo* proximity labeling for 2 h. After treatment, total cell extracts were prepared from the harvested cells. Biotinylation was checked on western blot, and cell extracts were snap‐frozen in liquid nitrogen and stored at −80 °C for subsequent mass spectrometry analysis (MS).

### Mass spectrometry

Total protein extracts (3 mg) were purified using Streptavidine magnetic beads (MACS^®^ Technology, Miltenyi: Köln, Germany) digested in column with trypsin, and analyzed in a single run on the mass spectrometer [43]. Quantitative proteomics combined with BAC TransgeneOmics reveals *in vivo* protein interactions [43]. The resulting tryptic peptide mixture was desalted prior to LC–MS/MS analysis on a C18 ZipTip (Omix C18 100 μL tips; Varian, Agilent Technologies: Santa Clara, CA, USA), and the purified peptide mixture was analyzed by LC–MS/MS using a nanoflow RP‐HPLC (LC program: linear gradient of 3–40% B for 100 min, solvent A: 0.1% formic acid in water, solvent B: 0.1% formic acid in acetonitrile) on‐line coupled to a linear ion trap‐Orbitrap (Orbitrap‐Fusion Lumos; Thermo Fisher Scientific) mass spectrometer operating in positive ion mode. Data acquisition was carried out in a data‐dependent fashion; the 20 most abundant, multiply charged ions were selected from each MS survey for MS/MS analysis (both spectra were acquired in the Orbitrap).

### MS data interpretation

Raw data were converted into peak lists using the in‐house proteome discoverer (v. 1.4) and searched against the Uniprot *Homo sapiens* database (downloaded 2019.6.12, 172 251 proteins) using our in‐cloud protein prospector search engine (v. 5.15.1, Thermo Fisher Scientific) with the following parameters: enzyme: trypsin with maximum two missed cleavage; mass accuracies: 5 p.p.m. for precursor ions and 10 p.p.m. for fragment ions (both monoisotopic); fixed modification: carbamidomethylation of Cys residues; variable modifications: acetylation of protein N‐termini; Met oxidation; cyclization of N‐terminal Gln residues, allowing maximum two variable modifications per peptide. Acceptance criteria: minimum scores: 22 and 15; maximum *E* values: 0.01 and 0.05 for protein and peptide identifications, respectively.

### Statistics

For densitometry analysis, western blot data were acquired using imagej software (Rasband, W.S., ImageJ, U.S. National Institutes of Health, Bethesda, MD, USA). The relative band densities were shown and normalized to an appropriate GAPDH band used as a reference protein. Results are presented as mean values ± SD and were compared using ANOVA with Tukey's multiple comparison *post hoc* test. Asterisks indicate a statistically significant difference from the appropriate control: *P < 0.05; **P < 0.01.

### Mathematical modeling

The regulatory network was converted to a set of nonlinear ordinary differential equations (ODEs) and analyzed using the techniques of dynamical system theory [44, 45, 46]. For details (i.e. for equations, codes, and software), see Supporting Information. Dynamical simulations were carried out using the program xppaut, which is freely available from http://www.math.pitt.edu/~bard/xpp/xpp.html [44, 45].

## Results

### The activation profile of ATF4 and its targets depends on ER stress level

In parallel with previously published data to further investigate the possible regulatory connections inside the UPR‐induced PERK branch, ER stress was induced in human embryonic kidney cells (HEK293T) using the most well‐known ER stressor, called thapsigargin (TG). TG disrupts the calcium homeostasis of the ER, resulting in ER stress [47]. In this study a proper TG concentration was used to mimic an acute but very severe ER stress in the cell. This ER stress induced by TG was successfully used in our previous studies [13, 14].

**Fig. 1:**
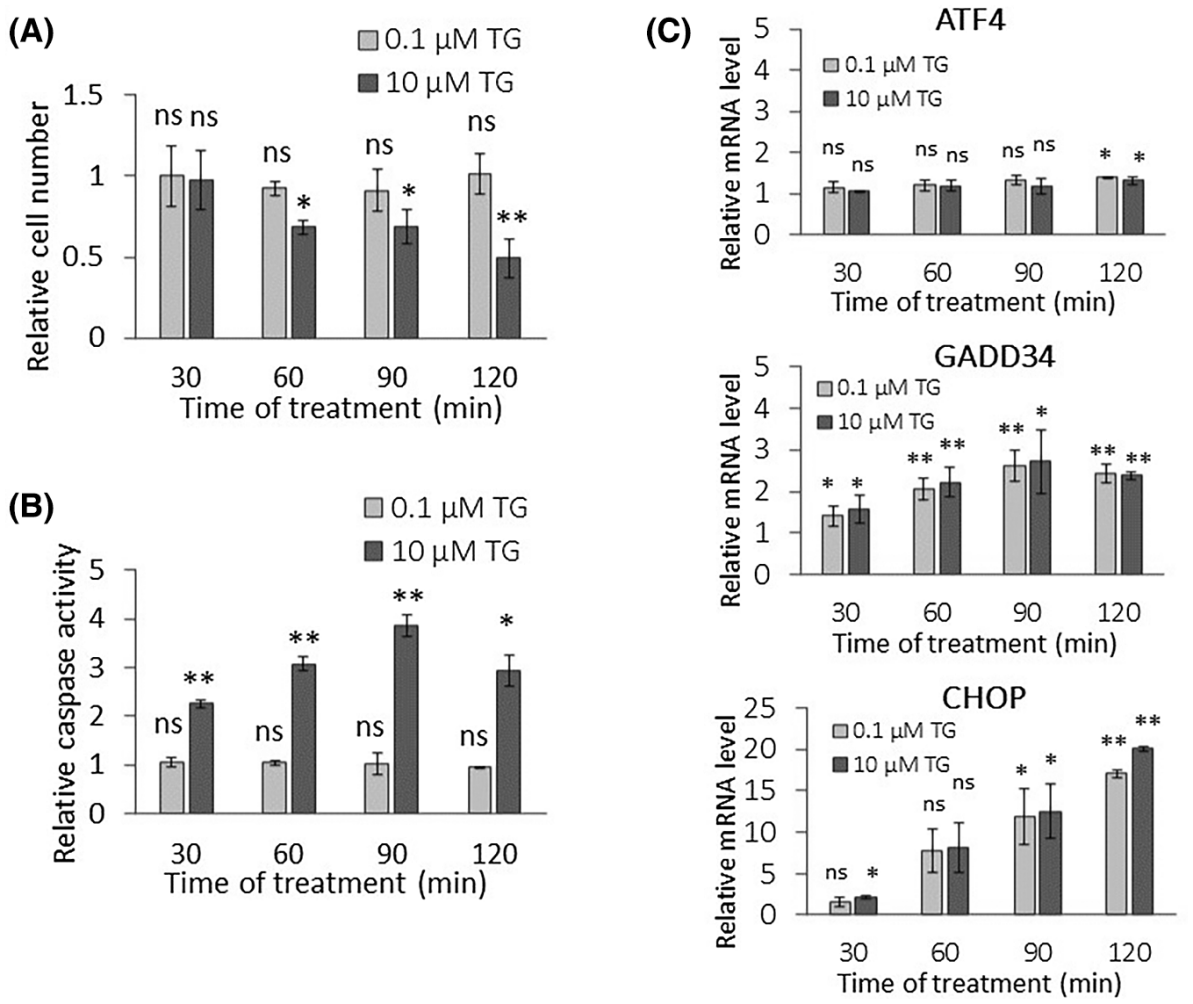
PERK pathway‐induced ER stress response mechanism depends on stress level. (A) The relative cell number and (B) the relative caspase 3/7 activity were followed in a case of low (light gray column, 0.1 μm thapsigargin for 2 h) and high (dark gray column, 10 μm TG thapsigargin for 2 h) levels of ER stress in HEK293T. (C) The mRNA level of key markers of PERK pathway (i.e. ATF4, CHOP, and GADD34) are depicted by real‐time PCR in case of low (light gray column, 0.1 μm thapsigargin) and high (dark gray column, 10 μm thapsigargin) level of ER stress in HEK293T for 2 h. Control samples were not treated with any reagent (including DMSO). For each of the experiments, three complete biological repeat experiments were carried out. Error bars represent standard deviation. In the calculation of statistical significance, all data were compared to the untreated controls. Results are presented as mean values ± SD and were compared using ANOVA with Tukey's multiple comparison *post hoc* test. Asterisks indicate statistically significant difference from the appropriate control: *P < 0.05; **P < 0.01. TG, thapsigargin.

First, both relative cell number and caspase activity were followed every 30 min for a 2‐h long period in response to a tolerable level (0.1 μm TG) or to an excessive level (10 μm TG) of ER stress (Fig. 1A,B). The low level of ER stress did not decrease significantly the relative cell number even after a 2‐h long treatment, while we found around a 50% decrease in the cell number after 2‐h long 10 μm TG treatment. Parallel to the drastic drop in cell number, intensive caspase activation was observed after 2‐h treatment with 10 μm, but not with 0.1 μm of TG, suggesting that cells were dying due to apoptotic cell death during an excessive level of ER stress.

**Fig. 2:**
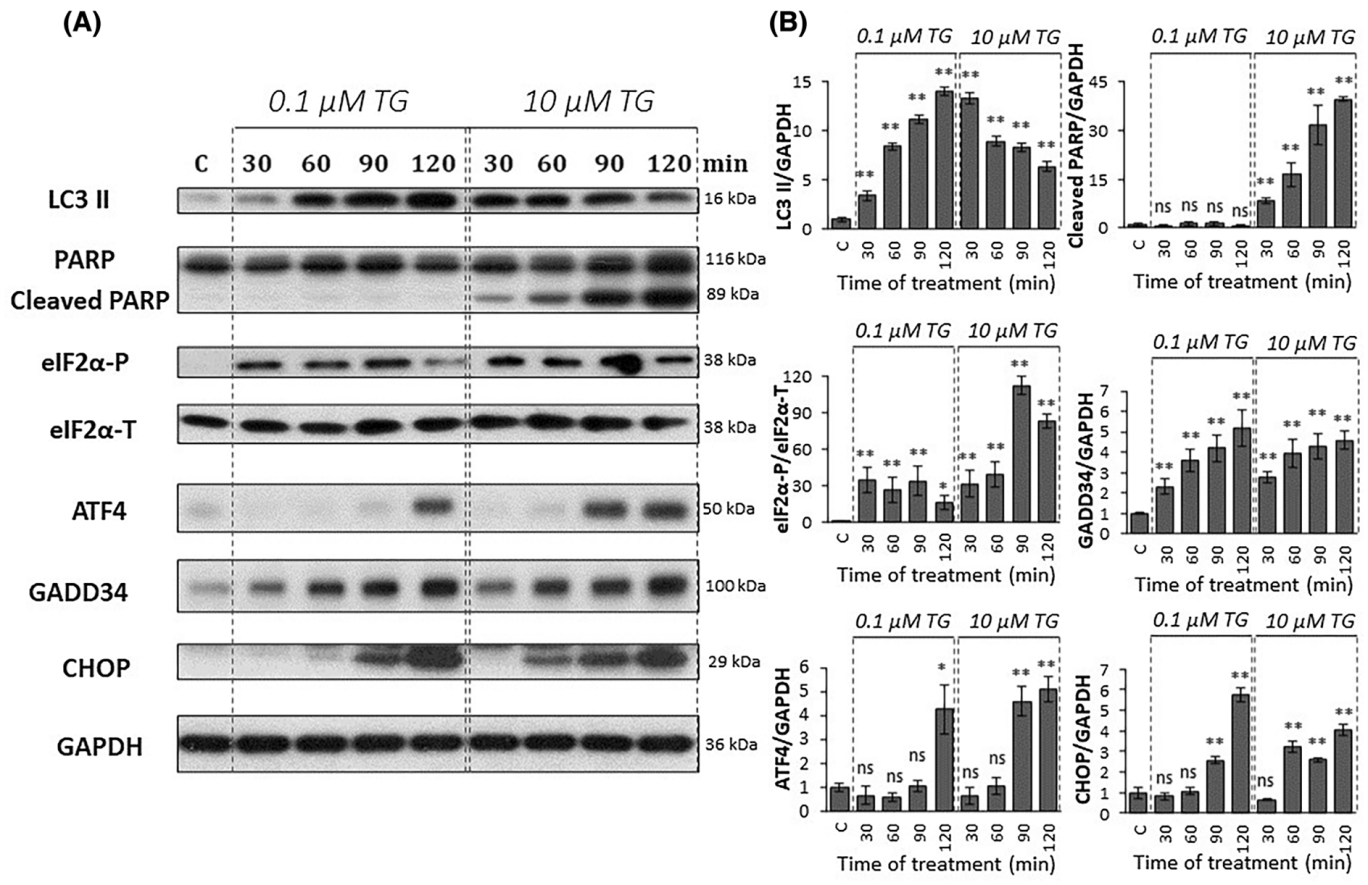
PERK pathway‐induced ER stress response mechanism depends on stress level. HEK293T cells were treated with low (0.1 μm thapsigargin for 2 h) and high (10 μm thapsigargin for 2 h) levels of ER stress and then the cells were denoted in time. (A) The markers of PERK pathway (i.e. eIF2α‐P, ATF4, CHOP, and GADD34) and the key markers of both autophagy (LC3) and apoptosis (PARP) were followed by immunoblotting. GAPDH was used as the loading control. The full‐length blots are included in the Supporting Information. (B) Densitometry data represents the intensity of cleaved LC3 II, PARP, ATF4, CHOP, and GADD34 normalized for GAPDH, while eIF2α‐P normalized for total level of eIF2α. Control samples were not treated with any reagent (including DMSO). For each of the experiments, three complete biological repeat experiments were carried out. Error bars represent standard deviation. In the calculation of statistical significance, all data were compared to the untreated controls. Results are presented as mean values ± SD and were compared using ANOVA with Tukey's multiple comparison *post hoc* test. Asterisks indicate statistically significant difference from the appropriate control: *P < 0.05; **P < 0.01. TG, thapsigargin.

Next, we tested how the key elements of the PERK pathway (i.e. ATF4, GADD34, CHOP) are affected at the mRNA and protein levels with respect to a low and high level of ER stress. The effect of both 0.1 and 10 μm TG treatment was followed during a 2‐h long time course in HEK293T cells, and samples were collected every 30 min (Figs. 1C and 2). Although the stress response mechanism seemed to be different depending on the applied ER stress level (i.e. apoptotic cell death was observed only at a high level of ER stress induced by 10 μm TG treatment), this difference could not be observed at either the mRNA or protein levels inside the PERK branch upon tolerable and nontolerable TG treatments. Namely, mRNA of ATF4 did not change significantly, while mRNA of GADD34 increased two‐fold upon ER stress treatments. In addition, the mRNA level of CHOP has shown a marked increase after 1‐h long treatment either at a low or high level of ER stress (Fig. 1C) as well. Similar results were observed when tunicamycin was used (Fig. S1).

In order to further explore the ER stress response mechanism, both tolerable and nontolerable ER stress events were checked at the protein levels by detecting the phosphorylation status of eIF2α in time (Fig. 2). While eIF2α showed a transient phosphorylation peak from 30 to 90 min at a low level of ER stress, its phosphorylated form remained significantly high during the 2‐h long TG treatment. The protein level of ATF4, GADD34, and CHOP increased during ER stress. However, the amount of both ATF4 and CHOP started to increase earlier during a high level of ER stress compared to a low level of ER stress (Fig. 2).

To further check the outcome of the cellular stress response mechanism, the key markers of autophagy (i.e. LC3II/GAPDH) and apoptosis (i.e. cleavage of PARP) were also followed in time (Fig. 2). At a low level of ER stress, the increasing ratio of LC3II/GAPDH was detected after 30 min of TG treatment, while PARP cleavage was not observed, suggesting that autophagy‐dependent survival could be active until the end of the TG treatment. However, with respect 10 μm TG treatment, cells showed only a transient increase of LC3II/GAPDH. This supposed decay of autophagy was followed by apoptotic cell death (see the significant PARP cleavage within 90 min of TG treatment in Fig. 2).

Our data suggest that the cellular life‐and‐death decision clearly depends on the ER stress level; however, significant differences cannot be observed in the activation of the profile of the key elements of PERK branch on either the mRNA or protein level.

### Inhibition of GADD34 results in downregulation of both ATF4 and CHOP during ER stress

**Fig. 3:**
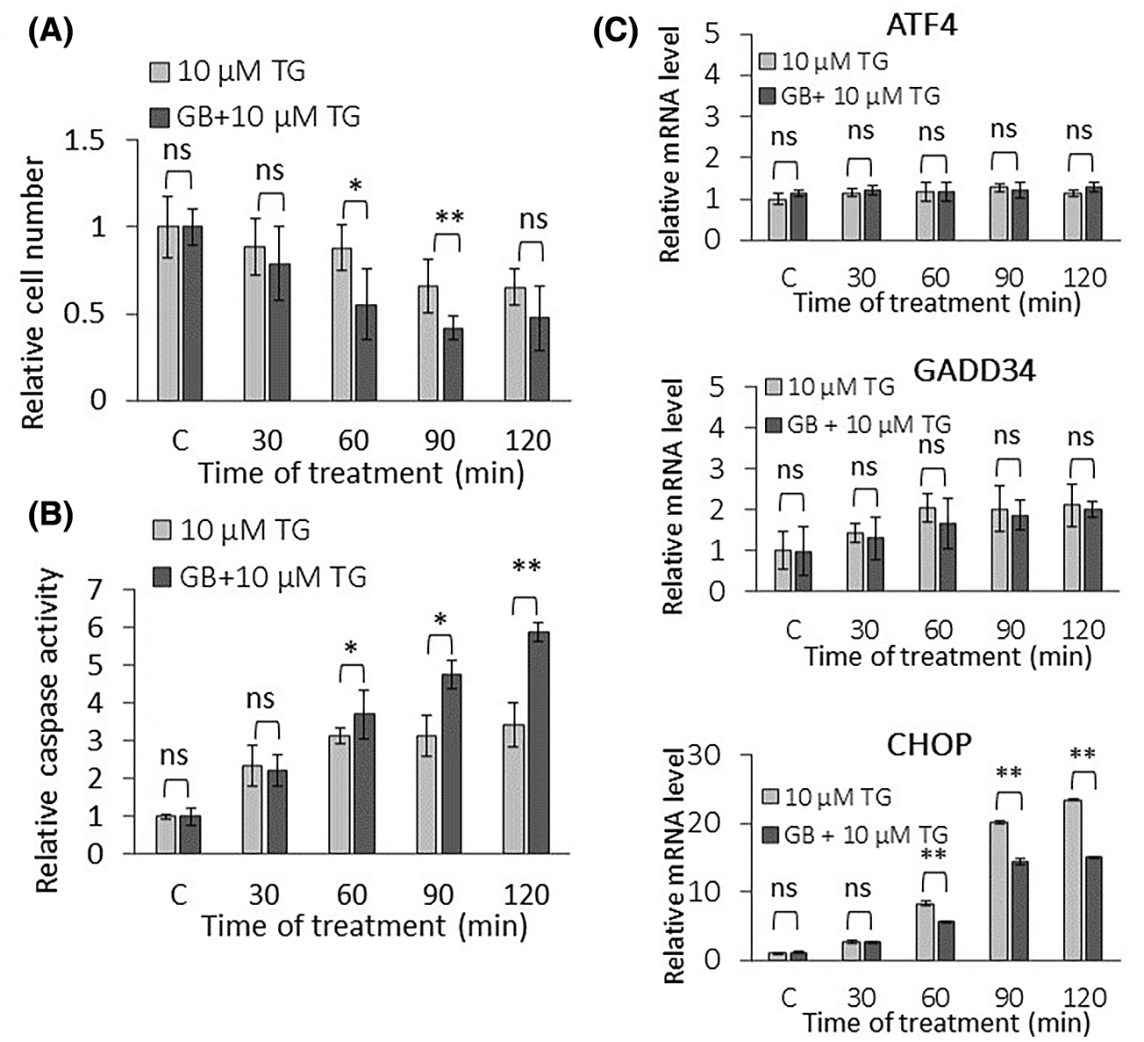
Excessive level of ER stress combined with inactivation of GADD34 affects PERK pathway on the mRNA level. (A) The relative cell number and (B) the relative caspase 3/7 activity were followed in the case of high level of ER stress (10 μm thapsigargin for 2 h) combined without (light gray column) or with (dark gray column) GADD34 inactivation via guanabenz treatment (50 μm for 1 h) in HEK293T. (C) GADD34 inactivation was inhibited by guanabenz treatment (50 μm for 1 h) followed by the addition of a high level of ER stress (10 μm thapsigargin for 2 h), then the cells were denoted in time. The mRNA level of key markers of PERK pathway (i.e. ATF4, CHOP, and GADD34) was depicted by real‐time PCR. Control samples were not treated with any reagent (including DMSO). For each of the experiments, three complete biological repeat experiments were carried out. Error bars represent standard deviation. In the calculation of statistical significance, data from guanabenz pretreated samples were compared to the not pretreated samples with the corresponding treatment time of 10 μm TG. Results are presented as mean values ± SD and were compared using ANOVA with Tukey's multiple comparison *post hoc* test. Asterisks indicate statistically significant difference from the appropriate control: *P < 0.05; **P < 0.01. GB, guanabenz; TG, thapsigargin.

**Fig. 4:**
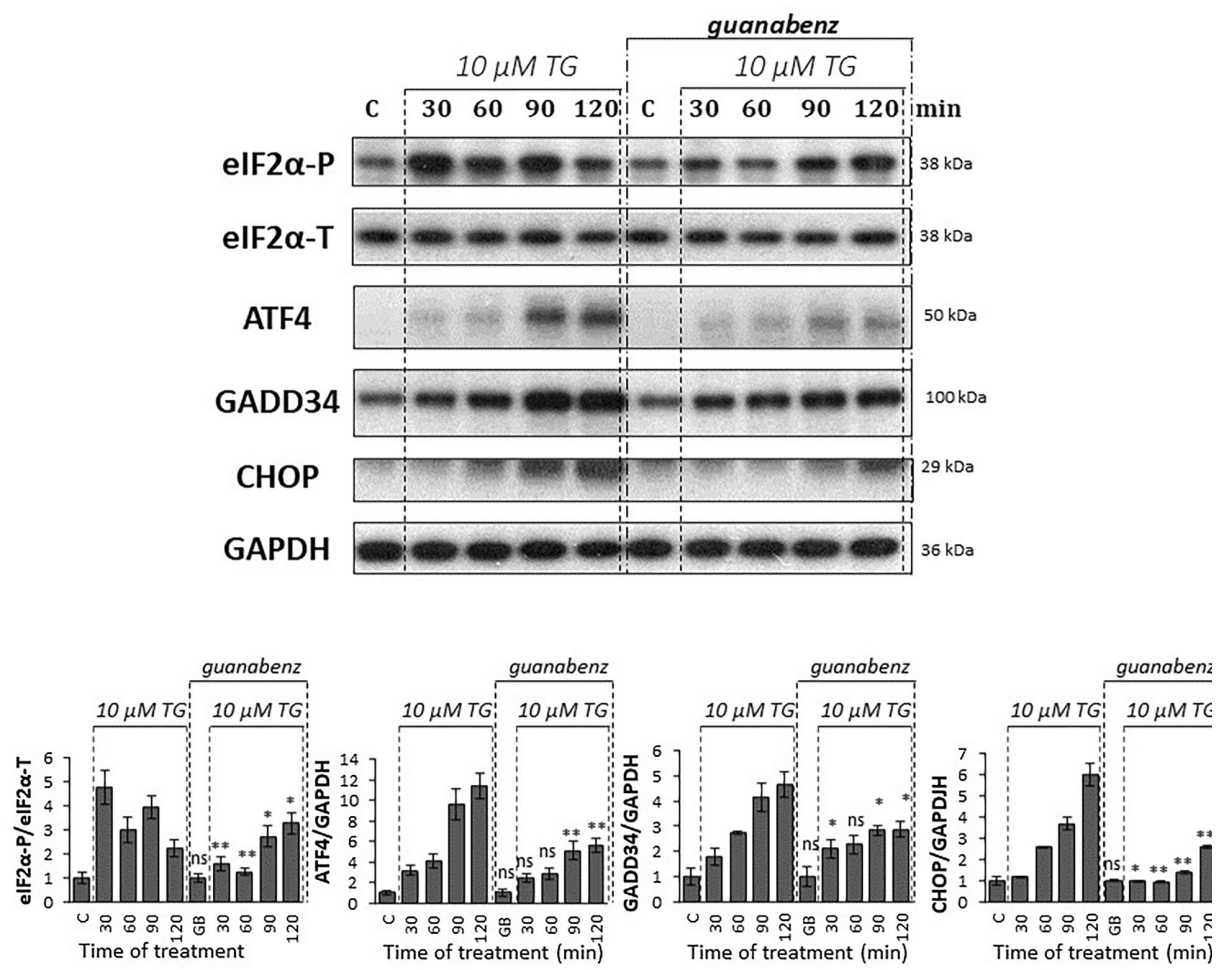
Excessive level of ER stress combined with inactivation of GADD34 affects PERK pathway on protein level. GADD34 inactivation via guanabenz treatment (50 μm for 1 h) was combined with a high level of ER stress (10 μm thapsigargin for 2 h) in HEK293T cells and then the cells were denoted in time. Control samples were not treated with any reagent (including DMSO). (upper panel) The markers of PERK pathway (i.e. eIF2α‐P, ATF4, CHOP, and GADD34) were followed by immunoblotting. GAPDH was used as the loading control. The full‐length blots are included in the Supporting Information. (lower panel) Densitometry data represent the intensity of ATF4, CHOP, and GADD34 normalized for GAPDH, while eIF2α‐P normalized for the total level of eIF2α. For each of the experiments, three complete biological repeat experiments were carried out. Error bars represent standard deviation. In the calculation of statistical significance, data from guanabenz pretreated samples were compared to the not pretreated samples with the corresponding treatment time of 10 μm thapsigargin (e.g. guanabenz pretreated control was compared to not pretreated control, and thapsigargin‐treated samples with the combination of guanabenz pretreatement were compared to the samples treated with thapsigargin for the same period of time). Results are presented as mean values ± SD and were compared using ANOVA with Tukey's multiple comparison *post hoc* test. Asterisks indicate statistically significant difference from the appropriate control: *P < 0.05; **P < 0.01. GB, guanabenz; TG, thapsigargin.

To further investigate the role of ATF4, GADD34k and CHOP in the ER stress response mechanism, a high level of ER stress was combined with inhibition of GADD34. First, HEK293T cells were pretreated with 50 μm of guanabenz for 1 h. Guanabenz (GB) is an α2 adrenergic receptor agonist and able to bind to GADD34/PP1 to inhibit it by disturbing the dephosphorylation of the α subunit of eIF2α [48]. GB treatment was followed by the addition of 10 μm TG and both relative cell number and relative caspase activity were checked every 30 min for 2 h (Fig. 3A,B). Samples pretreated with GB have shown a slight but significant decrease in the relative cell number after addition of 10 μm TG treatment compared to nonpretreated cells (Fig. 3A). The decrease in the relative cell number in GB pretreated cells was strictly associated with a slight, but significant, increase in caspase activity in a parallel manner (Fig. 3B). These results further confirm that addition of GADD34 inhibitor has a negative effect on cell survival in the case of ER stress; therefore, ATF4, GADD34, and CHOP were checked in HEK293T cells both at the mRNA and protein levels in the same experiment (50 μm GB for 1 h, then 10 μm TG for 2 h) (Figs. 3C and 4).

Partial downregulation of GADD34 activity by GB pretreatment has not changed the expression level of ATF4 and GADD34 mRNAs either (Fig. 3C), but induced a slight reduction of GADD34 at the protein level (Fig. 4), while both CHOP mRNA and protein levels showed a reduced increase in pretreated samples (Fig. 3). GB pretreatment maintained eIF2α phosphorylation longer compared to nonpretreated cells: P‐eIF2α showed an increasing trend even at 2 h of treatment. Thus, the effect of GB on the dephosphorylation of eIF2α by GADD34 was less pronounced, as the transient phosphorylation that was observed in the nonpretreated case at 120 min was not reduced compared to the 90 min 10 μm TG treatment. Since GB diminished the phosphatase activity of GADD34, therefore eIF2α‐P did not get dephosphorylated not even 2 h after TG addition (Fig. 4). Interestingly, ATF4 protein has shown a remarkable decrease on its western blot analysis in cells treated with both GB and TG, supporting that GADD34 had some positive effect on the ATF4 protein level. In addition, the significant decrease observed in CHOP both at mRNA and protein levels suggests that active GADD34 had some direct or indirect positive effect on CHOP too. In addition, silencing of GADD34 combined with thapsigargin‐ or tunicamycin‐induced ER stress showed similar results at the protein level in HEK cells, further confirming our above‐mentioned results (Figs. S2 and S3).

Guanabenz treatment might have side effects on the mTOR pathway, the key regulator of cellular homeostasis. In order to investigate whether GADD34 inhibition causes changes in mTOR activity, the mTOR pathway was monitored via its key substrates when silencing of GADD34 was combined with thapsigargin or tunicamycin treatment. The function of mTOR was followed by phosphorylation of the upper band of 4EBP1 (Thr37/46) and phosphorylation of p70 S6 kinase, respectively. Since neither 4EBP1 nor p70 phosphorylation changed significantly upon GADD34 silencing combined with thapsgiargin or tunicamycin treatment, we suggest that no changes in the mTOR was observed, compared to nonsilenced thapsighargin or tunicamycin treatments (Figs. S4 and S5).

These data assume that GADD34 has some positive effect on its own activation via ATF4 and it also upregulates CHOP upon an excessive level of ER stress.

### Silencing of CHOP combined with ER stress does not have any negative effect on ATF4.

**Fig. 5:**
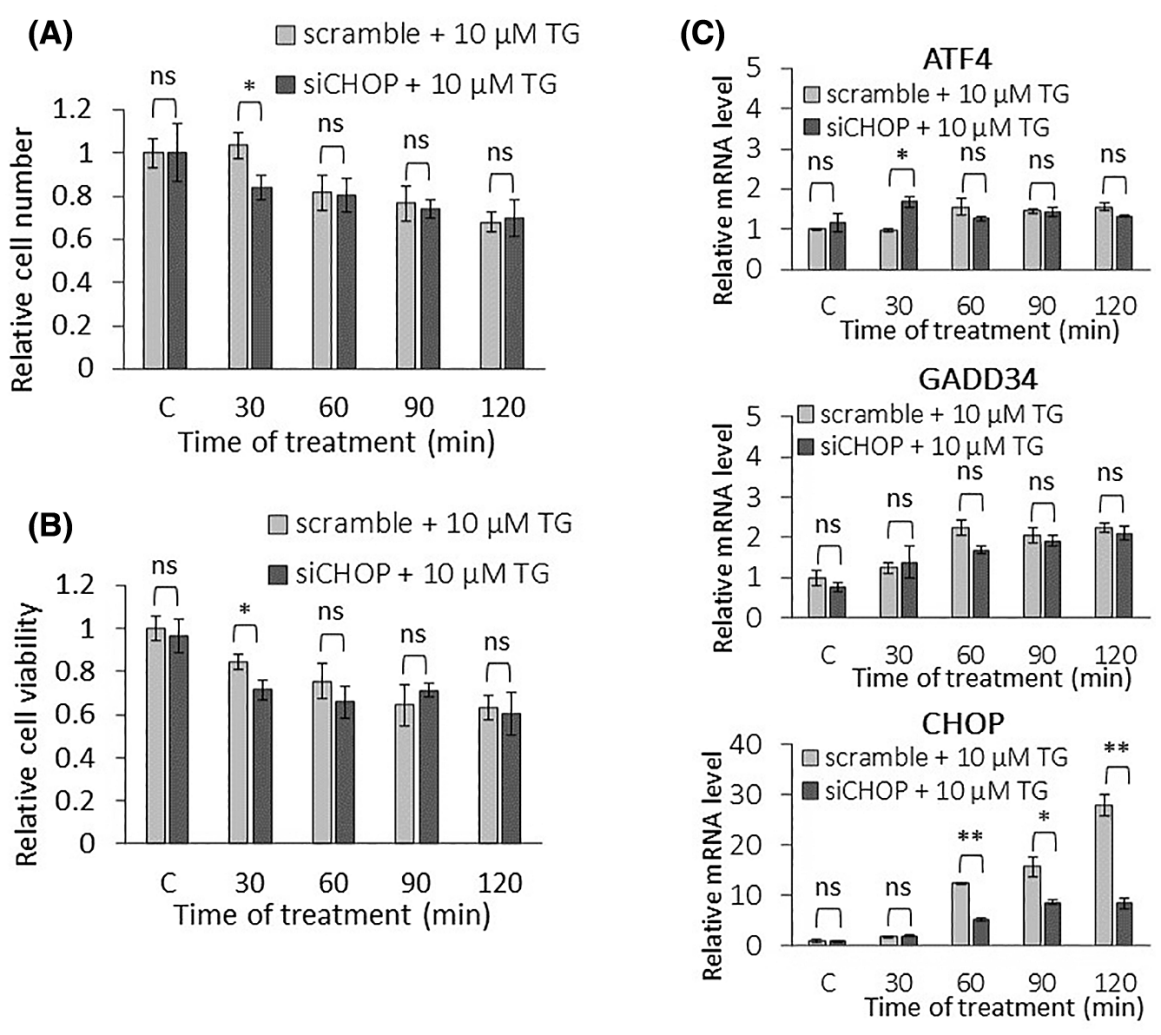
Excessive level of ER stress combined with CHOP silencing has a minor effect on the PERK pathway on the mRNA level. (A) The relative cell number and (B) the relative cell viability were followed in the case of a high level of ER stress (10 μm thapsigargin for 2 h) combined without (light gray column) or with (dark gray column) CHOP silencing in HEK293T cells. (C) CHOP was silenced, then a high level of ER stress (10 μm thapsigargin for 2 h) was induced and then the cells were denoted in time. The mRNA level of key markers of PERK pathway (i.e. ATF4, CHOP, and GADD34) was followed by real‐time PCR. Control samples were not treated with any reagent (including DMSO). For each of the experiments, three complete biological repeat experiments were carried out. Error bars represent standard deviation. In the calculation of statistical significance, data from CHOP‐silenced samples were compared to the nonsilenced samples with the corresponding treatment time of 10 μm thapsigargin (e.g. CHOP‐silenced control was compared to nonsilenced control, and thapsigargin‐treated samples with the combination of CHOP‐silencing were compared to the nonsilenced samples treated with thapsigargin for the same period of time). Results are presented as mean values ± SD and were compared using ANOVA with Tukey's multiple comparison *post hoc* test. Asterisks indicate statistically significant difference from the appropriate control: *P < 0.05; **P < 0.01. TG, thapsigargin.

**Fig. 6:**
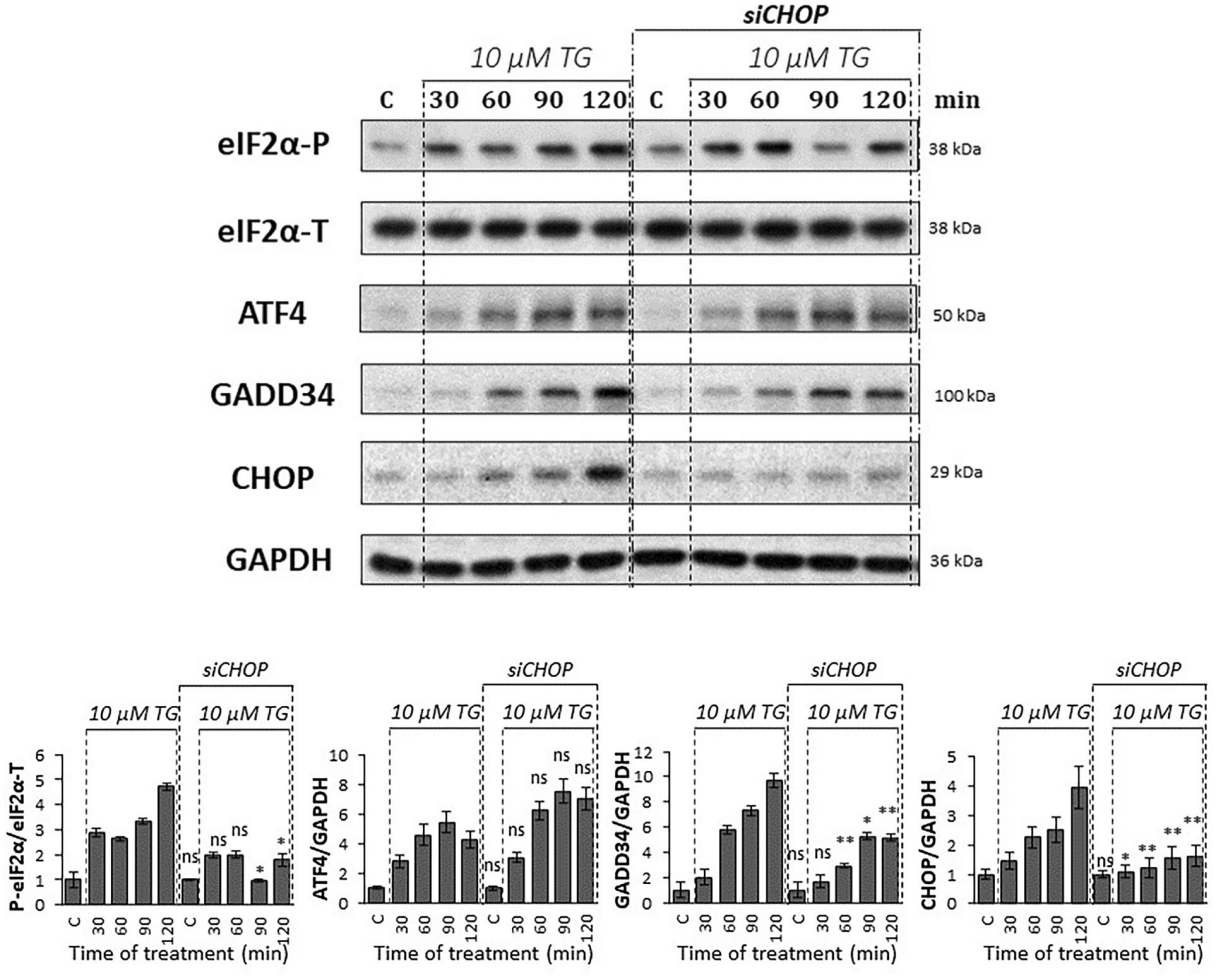
Excessive level of ER stress combined with CHOP silencing affects the PERK pathway on protein level. CHOP was silenced, then a high level of ER stress (10 μm thapsigargin for 2 h) was induced in HEK293T cells and then the cells were denoted in time. Control samples were not treated with any reagent (including DMSO). (upper panel) The markers of PERK pathway (i.e. eIF2α‐P, ATF4, CHOP, and GADD34) were followed by immunoblotting. GAPDH was used as the loading control. The full‐length blots are included in the Supporting Information. (lower panel) Densitometry data represent the intensity of ATF4, CHOP, and GADD34 normalized for GAPDH, while eIF2α‐P normalized for total level of eIF2α. For each of the experiments, three complete biological repeat experiments were carried out. Error bars represent standard deviation. In the calculation of statistical significance, data from CHOP‐silenced samples were compared to the nonsilenced samples with the corresponding treatment time of 10 μm thapsigargin (e.g. CHOP‐silenced control was compared to nonsilenced control, and thapsigargin‐treated samples with the combination of CHOP‐silencing were compared to the nonsilenced samples treated with thapsigargin for the same period of time). Results are presented as mean values ± SD and were compared using ANOVA with Tukey's multiple comparison *post hoc* test. Asterisks indicate statistically significant difference from the appropriate control: *P < 0.05; **P < 0.01. TG, thapsigargin.

To reveal the role of CHOP in the ER stress response mechanism, CHOP silencing was also studied, followed by the addition of the excessive level of the ER stressor. CHOP was directly silenced with siRNA in HEK293, and the efficiency of the silencing by siCHOP was tested both on the mRNA and protein levels (Figs. 5C and 6). Both relative cell number and viability was measured when transfection with siCHOP was followed by addition of 10 μm TG (Fig. 5A,B). No significant increase in either relative cell number or relative cell viability was observed, suggesting that during short TG treatment the positive effect of CHOP silencing on cell survival was not detectable.

To check the ER stress response mechanism, the phosphorylation status of eIF2α was tested by immunoblotting (Fig. 6). eIF2α‐P seemed to show a similar characteristic when 10 μm of TG was preceded with/without transfection of CHOP siRNA.

To further confirm the effect of CHOP silencing on the PERK pathway, the expression of CHOP mRNA was also followed, while the activation profile of both ATF4 and GADD34 were detected at the mRNA and protein levels (Figs. 5C and 6) as well. Although GADD34 was not affected at the mRNA level, CHOP silencing resulted in a significantly milder activation of the GADD34 protein than nonsilenced thapsigargin treatment. These data assume that CHOP acts positively on GADD34, but only at the protein level. Interestingly, neither mRNA nor protein expression of ATF4 was changed when CHOP silencing was followed by the addition of an excessive level of ER stressor.

Since CHOP silencing had not shown a significant change in the amount of ATF4 on the protein level, we investigated whether CHOP silencing was able to influence the transcriptional activity of ATF4. First, the activity of TRB3, another substrate of ATF4, was followed on the protein level, which was not inhibited by CHOP silencing in 10 μm TG treatment (Fig. S6). In addition, we examined whether ATF4 enters the nucleus during treatment, where it can be transcriptionally active. For this purpose, protein fractions of cytoplasm and nucleus were analyzed during 10 μm TM treatment combined with or without CHOP silencing by siRNA (Fig. S7). Our data confirmed that ATF4 entry into the nucleus was not inhibited by CHOP silencing at high levels of ER stress. These results suggest that the transcriptional activity of ATF4 is not affected by CHOP silencing.

To further examine whether the silencing of CHOP was able to affect other aspects of the stress response mechanism, the activity of the ISR nonrelated genes BiP and p58ipk were followed upon 10 μm TG treatment combined with or without CHOP silencing (Fig. S6). When thapsigargin treatment was combined with CHOP silencing, the activity of BiP and p58ipk was not increased significantly compared to the control, although both proteins appeared to be highly active in the CHOP‐silenced control.

Our data confirm that CHOP has some positive effect on GADD34, while the ATF4 level is not affected when CHOP is diminished upon a high level of ER stress.

### For proper ER stress the response mechanism extra feedback loops are suggested between ATF4 and its targets

The question immediately arises as to how it is possible to get various outcomes for ER stress response in the case of downregulation of CHOP or GADD34 combined with a short but excessive level of ER stress. This is really interesting since both of them are activated by ATF4, and CHOP has a positive effect on GADD34 too. Interestingly, the already experimentally suggested connections between ATF4, CHOP, and GADD34 [22, 30, 32, 33, 49] would assume the same results under our above‐mentioned experimental conditions (i.e. CHOP or GADD34 downregulation in the presence of a high level of ER stress). However, our novel experimental data assume extra crosstalk(s) in the control network. To explore this contradiction, a systems biological analysis was carried out. First a wiring diagram of a small regulatory network of the response mechanism was created to focus on the key components of the PERK branch of UPR (Fig. S8).

**Fig. 7:**
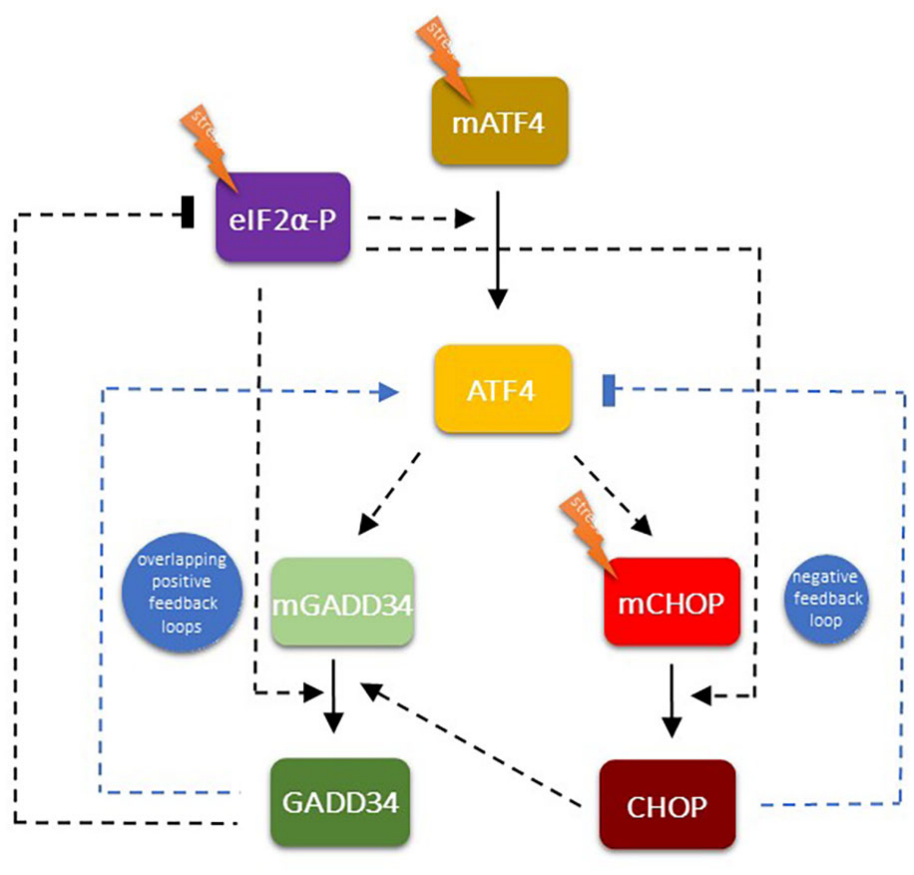
The wiring diagram of the regulatory network of the PERK pathway‐controlled ER stress response mechanism. The regulatory elements are denoted by isolated colored boxes. Dashed line shows how the components can influence each other, while blocked end lines denote inhibition. Previously established relationships are illustrated by black lines, while newly hypothesized (this study) relationships are shown with blue lines. Based on our molecular biological experiments and mathematical modeling, we propose new feedback loops within the PERK pathway: an extra positive feedback loop between GADD34 and ATF4, a negative feedback loop between CHOP and ATF4, and an indirect positive feedback loop between GADD34 and CHOP.

Upon ER stress, eIF2α gets activated by phosphorylation. Also, increasing the level of ER stress induces mRNA synthesis of ATF4. eIF2α‐P also promotes the translation of ATF4. These connections guarantee that ER stress induces ATF4 both transcriptionally and translationally. Active ATF4, as a transcription factor, induces the transcription of the mRNA of its targets, called GADD34 and CHOP, respectively. GADD34 and CHOP proteins get proportionally synthesized according to the level of GADD34 and CHOP mRNAs upon ER stress. CHOP also gets activated by increasing the level of ER stress. Already published scientific data have revealed that CHOP had a positive effect on GADD34, resulting in a coherent feedforward loop in the control network (Fig. S9). According to these regulatory connections, a simple mathematical model was generated (for details, see Supporting Information) and our experimental results (i.e. tolerable and excessive levels of ER stress, GADD3 or CHOP downregulation combined with a high level of ER stress) were checked by computer simulations (Figs. S10 and S11). With this simple model we were not able to reproduce our experimental data; therefore, we had to build in new connections into our simple model (Fig. 7).

**Fig. 8:**
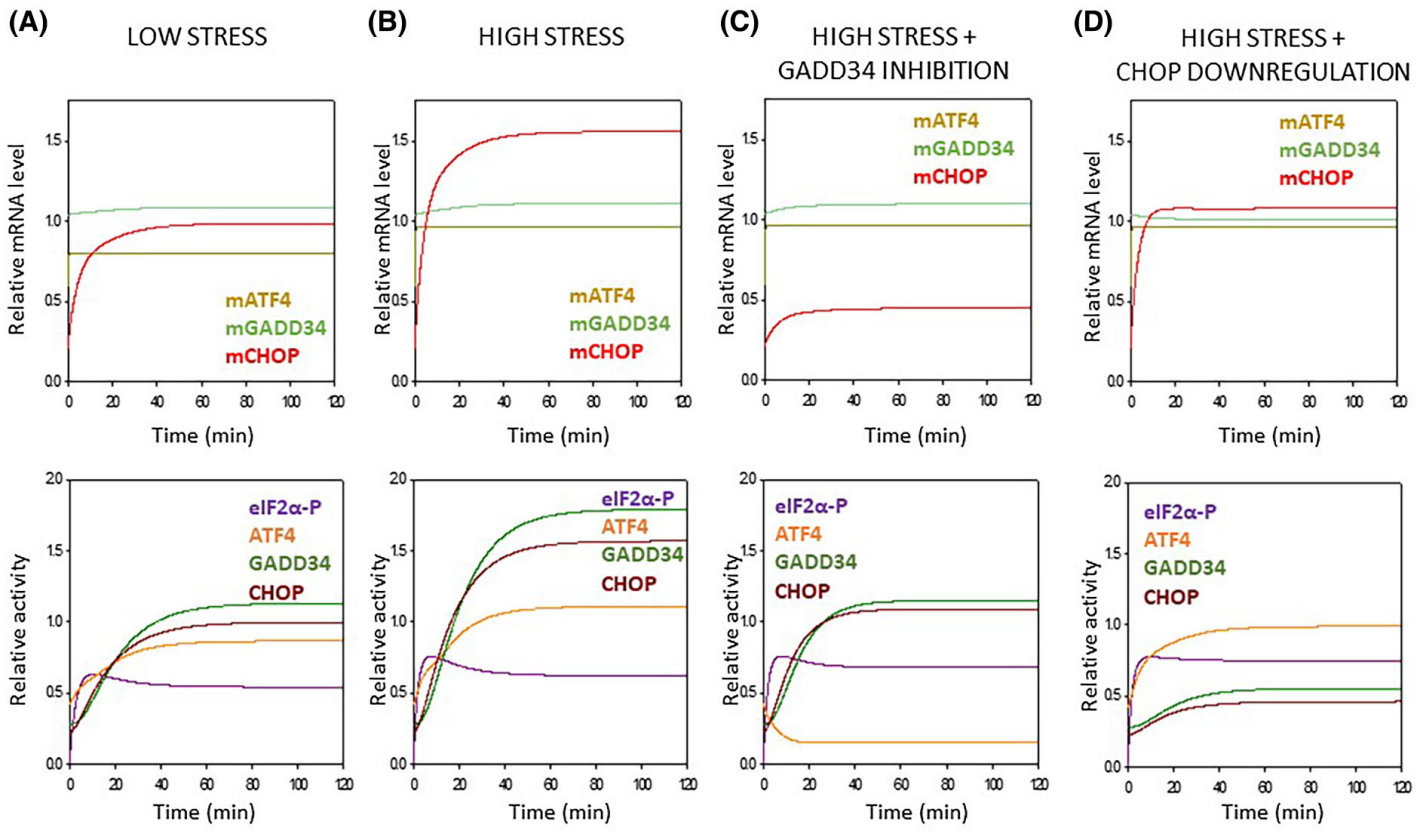
Computational analysis of the regulatory network of the PERK pathway‐controlled ER stress response mechanism. Numerical simulations of (A–D, upper panel) mRNA levels (i.e. mRNA of ATF4, CHOP, and GADD34) and (A–D, lower panel) protein activities (such as eIF2α‐P, ATF4, CHOP, and GADD34) during various ER stress events (about parameter values, see the code found in the Supporting Information). (A) The tolerable level of ER stress, while (B) refers to an excessive level of ER stress. A high level of ER stress was also combined (C) with guanabenz treatment or (D) CHOP silencing.

We claim that both GADD34 and CHOP have feedback effects on ATF4. Namely, GADD34 enhances the protein synthesis of ATF4, while CHOP has a negative effect on its activator generating positive and negative feedback loops, respectively (Fig. 7). To theoretically investigate the role of these feedback loops in the control network upon ER stress, the relative mRNA and protein levels were followed over time (Fig. 8).

If a tolerable or excessive level of ER stress was induced, CHOP shows a significant activation on the mRNA level. The mRNAs of both ATF4 and GADD34 were also increased two‐fold (Fig. 8).

GADD34 inhibition combined with a high level of ER stress results in an interesting phenotype due to the presence of feedback loops in the control network. Since GADD34 became inactivated via GB treatment, it could not promote the ATF4 anymore. Therefore, the mCHOP level became lower and all the three key regulators of PERK pathway (i.e. ATF4, GADD34, CHOP) showed a significant decrease in the protein level (Fig. 8).

Surprisingly (Fig. 6), we obtained an even more interesting effect when a high level of ER stress was combined with CHOP silencing. In that case, when the CHOP level was decreased by siRNA, the ATF4 level was not affected, although both GADD34 and CHOP decreased significantly at the protein level. Since CHOP has a negative effect on ATF4, in the absence of CHOP, ATF4 should be more active. However, CHOP silencing also diminished the CHOP → GADD34 connection from the control network, resulting in that GADD34 could not help the hyperactivation of ATF4. The diminishment of CHOP counteracted the effects of both positive and negative feedback loops, resulting in no change of either mRNA or protein levels of ATF4 (Fig. 8).

To investigate whether GADD34 affects directly or indirectly ATF4, we decided to investigate the GADD34 interactome to reveal its possible direct connection on the control network. In order to dissect this, we used the proximity labeling proteomic approach called TurboID [50]. HEK293T cells were transfected by GADD34‐TurboID chimeric protein and treated with 10 μm TG for 2 h in the presence of biotin. MS analysis of the cell extracts revealed many changes of the interactome in response to high stress treatment (Tables S1 and S2), but neither ATF4 nor CHOP was found to localize in the molecular proximity of GADD34, either in the presence or in the absence of TG.

Our theoretical analysis confirms the importance of the positive feedback loop between ATF4 and GADD34 and the negative feedback loop between ATF4 and CHOP in the presence of ER stress and are in accordance with the experimental data obtained.

## Discussion

One of the most important abilities of a living cell is to maintain its genetic integrity with the help of protein–protein control networks upon various stress events. Many scientific results have already revealed that endoplasmic reticulum stress is observed in several human diseases; therefore, understanding the dynamic characteristics of the ER stress response mechanism would be essential to understand these disorders [19, 51]. ER stress rapidly induces a complex signal transduction pathway, called unfolded protein response (UPR) [16].

In the last 10 years intensive systems biological studies were performed in our lab to investigate these aforementioned dynamic characteristics of the ER stress response mechanism. Using both experimental and theoretical methods we verified that UPR induces autophagy upon tolerable ER stress to enhance cell survival by self‐digesting damaged and/or harmful proteins in the cell; however, excessive levels of ER stress results in apoptotic cell death preceded by a transient peak of autophagy [13, 14, 15]. Here we further confirm that a short or so‐called “acute” ER stress induced by thapsigargin might result in autophagy or apoptosis, depending on the concentration of the ER stressor (Figs. 1 and 2). This concentration of TG (i.e. 10 μm for 2 h) represents a transient treatment with a very high level of the ER stressor. In parallel with the literature data [6], we have recently shown that a double negative feedback loop between the main controllers of autophagy and apoptosis inducers ensures the switch‐like activation of the suicide mechanism, guaranteeing a precise decision‐making process between life and death if the ER stress reaches a critical value (see the dynamical characteristic of autophagy and apoptosis in Fig. 8A,B).

We also started to investigate the exact role of the key molecular components of UPR in this process. With the experimental data from Deegan *et al*. [20] we proved the presence of a positive feedback loop between IRE1 and PERK [14]. The systems biological analysis performed in our laboratory revealed that this crosstalk between these two branches of UPR has an essential role in ensuring the irreversible characteristic of apoptosis induction upon nontolerable ER stress [14]. This positive feedback loop of UPR assures the point‐of‐no‐return characteristic of the suicide mechanism if the stress level reaches a critical value. The question immediately arises, whether extra positive feedback loops are present inside the different branches of UPR generating an even more robust irreversible switch upon an excessive level of ER stress. Although all the three branches of UPR got activated upon a high level of ER stress, our research here was focused on the dynamic characteristics of the key elements of the PERK pathway―as the most intensively studied branch of the UPR such as ATF4 and its targets, called GADD34 and CHOP. It has been shown that CHOP had a positive effect on GADD34 [32, 33], while GADD34 downregulation has been suggested to have a negative effect on CHOP activation upon cellular stress [52, 53, 54, 55, 56]. Until now, there were no exact data on whether GADD34 can promote CHOP directly or indirectly upon ER stress.

To understand this question, an excessive level of ER stress was combined with downregulation of either GADD34 activity or CHOP expression and the amount of ATF4, GADD34, and CHOP detected both at the mRNA and protein levels (Figs. 3, 4, 5, 6). Inhibition of GADD34 followed by TG treatment resulted in a significant decrease in the amount of CHOP mRNA and protein (Figs. 3, 4 and 8C), confirming that GADD34 had a positive effect on CHOP. Besides, the protein level of ATF4 also displayed a significant decrease during combined treatment, suggesting that GADD34 might act positively but indirectly on CHOP via ATF4. We are the first to show that this positive feedback loop is present inside the PERK branch of UPR upon ER stress; however, the exact molecular mechanism of this connection needs to be further clarified in the future.

Depletion of CHOP by silencing it with CHOP siRNA resulted in a very interesting phenotype when an excessive level of ER stress was induced for a short time in a human cell line. Namely, GADD34 and ATF4 were not changed on their mRNA levels at all. At the same time, the GADD34 protein level got lower, while the ATF4 protein level was not affected (Figs. 5 and 6), suggesting that CHOP and GADD34 were regulated by post‐transcriptional rather than transcriptional. However, it has previously shown that knockdown of CHOP combined with permanent induction of ER stress was able to downregulate GADD34 on the mRNA level [32, 33]. These contradictory results between transient and permanent treatments with an excessive level of ER stressor needs further investigation in the future.

These abovementioned unexpected findings cannot be explained by a sole indirect positive feedback loop between GADD34 and CHOP via ATF4; otherwise, the ATF4 level should decrease when GADD34 became downregulated during ER stress. Our theoretical analysis nicely revealed (Figs. 7 and 8) that this phenotype (Figs. 5 and 6) could only be observed if there was an extra feedback loop in the network. We claim that beside its positive effect on ATF4 via GADD34 positive (ATF4 → GADD34 → ATF4), CHOP has to inhibit ATF4 generating an extra, negative (ATF4 → CHOP 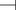 ATF4) feedback loop in the control network. When CHOP was inhibited upon ER stress, GADD34 had a lower level due to the absence of the CHOP → GADD34 connection. Although ATF4 got released from the CHOP‐dependent inhibitory effect, since the GADD34 level was low, no significant increase of the ATF4 level was observed (Figs. 7 and 8). Interestingly, the GADD34‐dependent negative effect on eIF2α‐P did not seem as effective upon a short but excessive level of ER stress (see Fig. 6, where eIF2α‐P did not increase significantly when CHOP silencing was combined with ER stress compared to TG treatment). We suppose that reduction of the acute ER stress response mechanism is controlled mainly by the CHOP 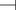 ATF4 feedback loop, while GADD34 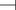 eIF2α‐P has an important role in permanent ER stress. However, the importance of these negative feedback loops (aka, GADD34 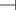 eIF2α‐P, and CHOP 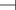 ATF4) at various ER stress levels needs to be further study.

Our interactome data showed that neither GADD34‐ATF4 nor CHOP‐GADD34 loops are based on direct interactions of the proteins. Since TurboID labels proteins in molecular proximity [57], and we found 118 biotinylated proteins annotated to UPR response, autophagy or apoptosis by Gene Ontology analysis (http://geneontology.org/), our results make it very unlikely that GADD34 would act on ATF4 or CHOP directly, or vice‐versa. Future experiments are needed to reveal the exact pathways involved in our theoretical feedback loops that we assumed based on our experimental data.

It has also shown that either CHOP or GADD34 overexpression reduced cell viability and resulted in apoptotic cell death upon cellular stress [30, 58, 59]. To investigate how our above‐mentioned novel feedback loops of the control network would affect ER stress response mechanisms, computational simulations were carried out (Fig. S11). The overproduction of either GADD34 or CHOP resulted in a significant increase of CHOP activity upon an excessive level of ER stress. We claim that either CHOP or GADD34 overexpression upon ER stress can be explained with our newly proposed feedback loops (i.e. ATF4 → GADD34 → ATF4 and ATF4 → CHOP 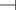 ATF4) of the PERK pathway.

Our previous theoretical analyses have underlined the importance of a positive feedback loop between GADD34 and CHOP via ATF4 to guarantee the irreversible switch‐like feature of the control system, but the question arises, what could be the role of the ATF4 → CHOP 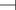 ATF4 negative feedback loop. When a target molecule acts back negatively to its inducer it always has a downregulating effect on a signal transduction pathway. Similar regulatory connection was characterized with Dr. Tamás Korcsmáros, when we proved that NRF2 had a negative feedback on its activator, called AMPK [60, 61]. Our analysis has indicated that this negative feedback loop has an essential role in preventing the hyperactivation of autophagy induced by both AMPK and NRF2 [62]. Similar to this observation, here we also suggest that CHOP blocks the hyperactivation of ATF4 upon an excessive level of ER stress. The proper balance between autophagy and apoptosis depends on the fine‐tuning of GADD34 and CHOP controlled feedback loops during the decision‐making process. The coordinated behavior of the positive and negative feedback loops guarantees the switch‐like characteristic depending on the level of ER stress. We suppose that both GADD34 and CHOP are essential to ensure the irreversible switch‐like feature of cellular life‐and‐death decisions.

Our mathematical model was directly based on results from the literature and our own experimental results. Since the experimental work was performed on only one cell line, HEK293T, we need to consider that the results are specific to this cell line. Although our new results together with the already published data suggest that our model might have a general regulatory mechanism in most human cell lines, this study has some limitations. For example, it is still necessary to investigate the ATF4‐GADD34‐CHOP regulatory triangle in the future, either in primary cell lines or *in vivo*. In addition, it is worthwhile to investigate the process with other ER stressors (such as DTT) in the future.

Among many others, ER stress is involved in a fatal disorder called inflammatory bowel disease (IBD) [63]. The newly verified “inter‐ and intramolecular crosstalks” of UPR and the systems‐level feedback loops influencing life‐and‐death decisions upon ER stress might bring us closer to understand the dynamical features of IBD. In the light of the new findings presented here, our plan is to successfully postpone the symptoms of IBD (e.g. by the addition of various autophagy inducers, such as resveratrol [64], rapamycin [65]). A precisely chosen concentration of these drugs might have a therapeutic role in the future to expand the lifespan of people suffering from IBD.

## Conflict of interest

The authors declare no conflicts of interest.

## Author contributions

OK and GB are involved in conceptualization; OK and GB are involved in methodology; MM is involved in experimental study; OK, MM, and NG are involved in investigation and resources; EÉK, KK, and NG are involved in TurboId and cloning; AP‐S involved in MS measurement and data analysis; OK, MM, NG, and AP‐S are involved in original draft preparation, review and editing; OK, MM, and NG are involved in visualization. All authors have read and agreed to the published version of the article.

## Supporting information


**Fig. S1.** Tunicamycin‐induced ER stress decreases cell number and turns on PERK‐pathway on mRNA level.
**Fig. S2.** Excessive level of ER stress (10 μM TG) combined with RNA silencing of GADD34 affects PERK pathway on protein level.
**Fig. S3.** Excessive level of ER stress (35 μM TM) combined with RNA silencing of GADD34 affects PERK pathway on protein level.
**Fig. S4.** Excessive level of ER stress (10 μM TG) combined with RNA silencing of GADD34 does not affect mTOR on protein level.
**Fig. S5.** Excessive level of ER stress (35 μM TM) combined with RNA silencing of GADD34 does not affect mTOR on protein level.
**Fig. S6.** The effect of CHOP silencing combined with excessive level of ER stress (10 μM TG) on ATF4 substrate TRB3 and ISR nonrelated ER stress marker BiP and p58ipk.
**Fig. S7.** CHOP silencing does not affect on the translocation of ATF4 to the nucleus during excessive level of ER stress (10 μM TG).
**Fig. S8.** The wiring diagram of regulatory network of PERK pathway controlled ER stress response mechanism when GADD34‐ATF4 positive and CHOP‐ATF4 negative feedback loops are not present.
**Fig. S9.** Computational analysis of regulatory network of PERK pathway controlled ER stress response mechanism when GADD34‐ATF4 positive and CHOP‐ATF4 negative feedback loops are not present.
**Fig. S10.** Computational analysis of regulatory network of PERK pathway controlled ER stress response mechanism when GADD34‐ATF4 positive and CHOP‐ATF4 negative feedback loops are not present.
**Fig. S11.** Computational analysis of regulatory network of PERK pathway controlled ER stress response mechanism when GADD34‐ATF4 positive and CHOP‐ATF4 negative feedback loops are present.
**Table S1.** The first 25 proteins showing decrease in their proximity‐dependent biotinylation in response to thapsigargin treatment.
**Table S2.** The first 25 proteins showing increase in their proximity‐dependent biotinylation in response to thapsigargin treatment.
**Appendix S1.** Basics to build up mathematical models.
**Appendix S2.** Mathematical codes for computational simulations.
**Appendix S3.** Original blots of figs.
**Appendix S4.** Efficiency of qPCR primers.

## Data Availability

The MS data presented in this study are openly available in FigShare at https://doi.org/10.6084/m9.figshare.19258859.
