# Supplementary material for: A systems biological analysis of the ATF4‐GADD34‐CHOP regulatory triangle upon endoplasmic reticulum stress
Source: FEBS Open Bio. 2022 Sep 27;12(11):2065–82. doi: 10.1002/2211-5463.13484 (PMC9623533; doi:10.1002/2211-5463.13484)
Supplement: Supplementary file 1 — Fig. S1. Tunicamycin‐induced ER stress decreases cell number and turns on PERK‐pathway on mRNA level. Fig. S2. Excessive level of ER stress (10 μM TG) combined with RNA silencing of GADD34 affects PERK pathway on protein level. Fig. S3. Excessive level of ER stress (35 μM TM) combined with RNA silencing of GADD34 affects PERK pathway on protein level. Fig. S4. Excessive level of ER stress (10 μM TG) combined with RNA silencing of GADD34 does not affect mTOR on protein level. Fig. S5. Excessive level of ER stress (35 μM TM) combined with RNA silencing of GADD34 does not affect mTOR on protein level. Fig. S6. The effect of CHOP silencing combined with excessive level of ER stress (10 μM TG) on ATF4 substrate TRB3 and ISR nonrelated ER stress marker BiP and p58ipk. Fig. S7. CHOP silencing does not affect on the translocation of ATF4 to the nucleus during excessive level of ER stress (10 μM TG). Fig. S8. The wiring diagram of regulatory network of PERK pathway controlled ER stress response mechanism when GADD34‐ATF4 positive and CHOP‐ATF4 negative feedback loops are not present. Fig. S9. Computational analysis of regulatory network of PERK pathway controlled ER stress response mechanism when GADD34‐ATF4 positive and CHOP‐ATF4 negative feedback loops are not present. Fig. S10. Computational analysis of regulatory network of PERK pathway controlled ER stress response mechanism when GADD34‐ATF4 positive and CHOP‐ATF4 negative feedback loops are not present. Fig. S11. Computational analysis of regulatory network of PERK pathway controlled ER stress response mechanism when GADD34‐ATF4 positive and CHOP‐ATF4 negative feedback loops are present. Table S1. The first 25 proteins showing decrease in their proximity‐dependent biotinylation in response to thapsigargin treatment. Table S2. The first 25 proteins showing increase in their proximity‐dependent biotinylation in response to thapsigargin treatment. Appendix S1. Basics to build up mathematical models. Appendix S2. Mathemati [file FEB4-12-2065-s001.docx]

**Supplementary Information**

**“A systems biological analysis of ATF4-GADD34-CHOP regulatory triangle**

**upon endoplasmic reticulum stress”**

**I. Basics to build up mathematical models**

*The schematic picture of our systems biological approach*

Systems biological approach means that we are use both theoretical and molecular biological tehcniques to understand dynamical charactersitc of the cellular stress reponse mechanism. We are creating experimental data, while the modelling approach we present here tries to explain the characteristic features of the control network. We also use already existing experimental data, either from the published papers or from databases containing referenced information. Then these data are used to create a static wiring diagram and it is explained by differential equations to capture the dynamics of the system. The models are then used to simulate the effect of the different elements.


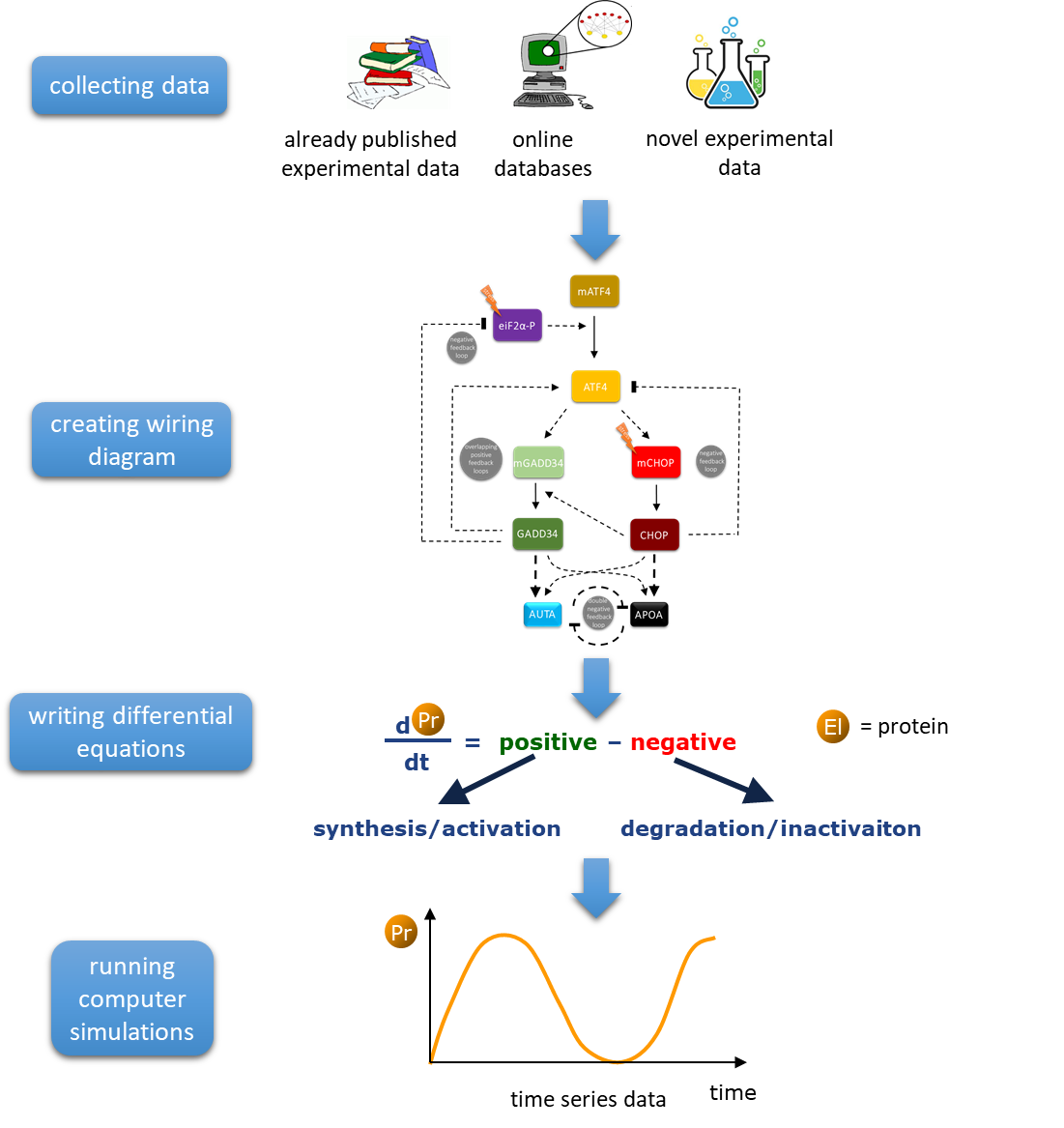


Pr

*General details about building up mathematical models*

In this section, we briefly describe the mathematical approach used to study the dynamical characteristic of the control network. A system level view can be developed by bringing together the components and interactions reported in the literature. Such a network can be translated into a set of mathematical equations that describe how each component concentration/activity in the network changes with the time. The rate of change of a component is described by ordinary differential equation (ODE) based on biochemical reaction kinetics (see equation below). Each biochemical reaction is represented as a term on the right hand side of the ODE for a component participating in the reaction (1, 2). Each reaction in the network can be described either by using law of mass action or Michaelis-Menten kinetics (3-5).

A generic differential equation describing the temporal changes of protein X_a_ is composed of two parts: production and consumption terms.

dX_a_/dt = k_s_ + k_act_*(X_T_-X_a_) – (k_d_ + k_in_)*X_a_

Where:

X_a_ – concentration of active X

X_T_ – total concentration of X

k_s_ – synthesis rate constant of X

k_act_ – activation rate constant of X_a_

k_d_ – degradation rate constant of X

k_in_ – inactivation rate constant of X_a_

The production can be given by protein synthesis and/or an activation term, while the consumption can be given by protein degradation and/or inactivation term. Usually synthesis, degradation, binding and dissociation reactions are described by mass action kinetics, whereas protein activity can be described either by mass action or Michaelis-Menten kinetics (3, 6). For example, if the activity of protein is controlled by covalent modification involving multi-site phosphorylations, Michaelis-Menten kinetics provides a good approximation for the process (7, 8). The value of parameters (rate constants, Michaelis constants) and initial conditions have to be specified in order to solve ODEs. The non-linear nature of biological processes makes it difficult to find the solution of ODEs analytically hence the equations has to be solved numerically. The equations can be solved using different numerical integration methods that are implemented as solvers in many of the freely available computer software.

Solving a set of non-linear ODEs gives the time evolution of the protein concentration/activity called **time courses**. Further, ODEs can be solved to obtain the input-output relationship called as **signal response curves** or as **one parameter bifurcation diagram** (2, 3, 9). An input is the signal strength that is varied to obtain the steady state behaviour of the control system. This helps to capture the qualitative changes in the behaviour of the system. For example, the system behaviour can become abrupt and discontinuous when signal strength is increased from a low value to high value. A point at which such a qualitative change in the system occurs is defined as bifurcation point (2).

*Building up the model of ER stress response mechanism*

Life‐and‐death decision induced by endoplasmic reticulum (ER) stress is directly regulated by the three pathways of UPR for short, called PERK, IRE-1 and ATF6. Here we focus on the key regulatory components of PERK pathway (i.e. eiF2a-P, ATF4, GADD34 and CHOP) to explain the cellular life‐and‐death decision upon ER stress, and therefore explore the key feedback loops of the control network. Our goal is to build up the simplest mathematical model by finding the key crosstalks inside the PERK branch of UPR. In this analysis we do not concentrate on a given protein itself, rather we try to figure out which are the essential regulatory motifs (i.e. feedback loops) of the control network to give that kind of answer what we have been already observed in previous experimental results and our novel data.

The detailed wiring diagram of our control network:


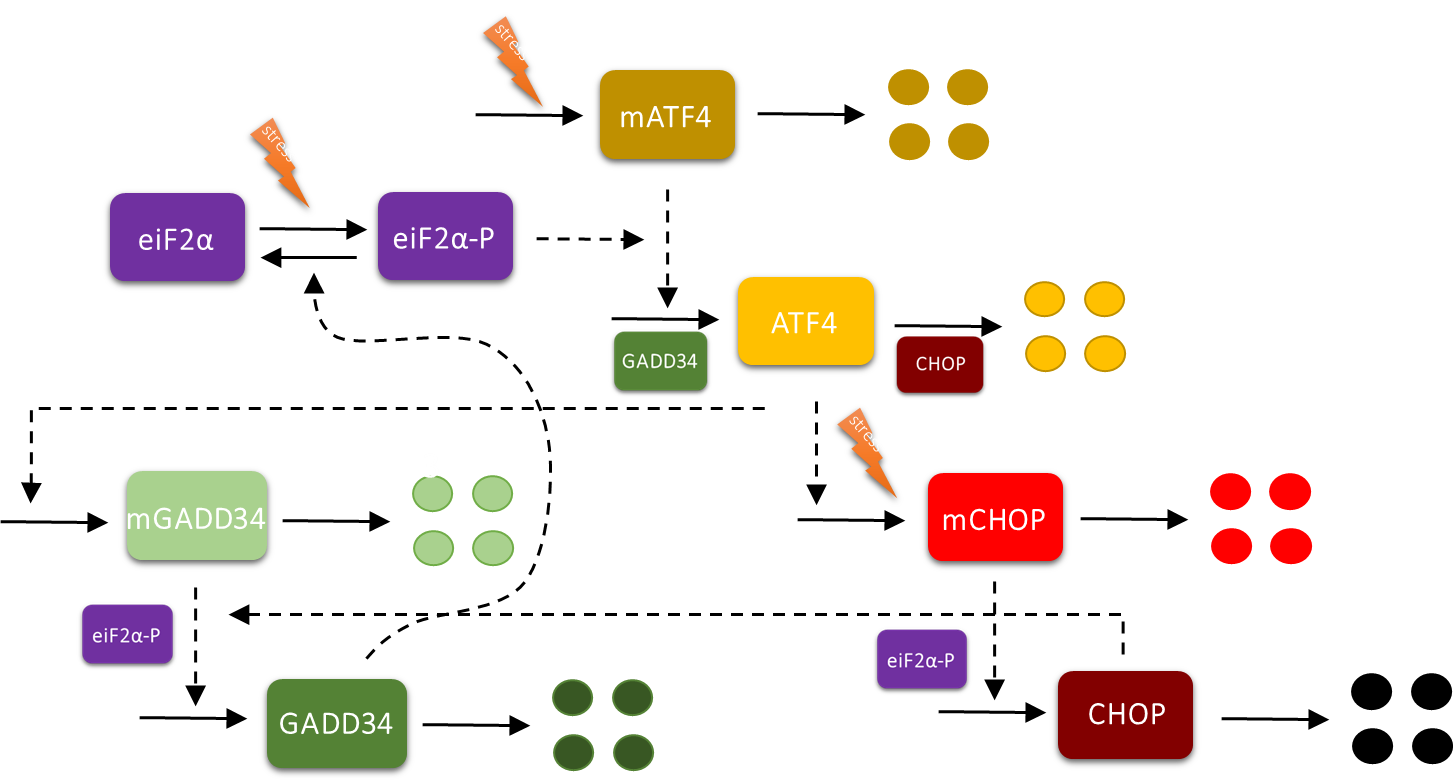


The regulatory elements are denoted by isolated coloured boxes. Dashed line shows how the components can influence each other, while blocked end lines denote inhibition. “Lightening” named “stress” refers where the ER stress acts on out wiring diagram.

The main assumptions and the limitations of the proposed models are the followings:

1. UPR with its three branches is so complex containing so many redundant pathways and regulatory cascades. Since we focus only on the feedback loops inside the PERK branch, we did not build in all the molecules of UPR network separately. Rather, only those elements of the PERK pathway are included in the model, which have important roles in determining the dynamical features of the ER stress response mechanism (i.e. eiF2-p, ATF4, CHOP and GADD34).
2. Since the activity of ATF4, CHOP and GADD34 was followed both on mRNA and protein level upon ER stress, therefore both the protein and mRNA forms of ATF4, CHOP and GADD34 are introduced in the model.
3. BIP/Grp78 and PERK are not included, we assume that ER stressors directly induce mATF4, eiF2a-P and mCHOP.
4. ER stress is introduced as a parameter (called “S”) and depends on its value to types of ER can be distinguished called low and high level of ER stresses, respectively. Low level of stress (S=5) always means tolerable stress, when the cell does not commit suicide, rather induces autophagy-dependent processes via UPR. Meanwhile high level of stress (S=30) refers to excessive level of ER stress, which promotes apoptotic cell death induced by the UPR. Under physiological conditions ER stress is zero (S = 0).
5. Parameter values are estimated on that way to investigate how the experimentally proved features of the control network can be captured by various feedback loops. The model allows us to make predictions that we can later verify experimentally. Since exact data about our parameters cannot be found in the literature (i.e. amount of UPR mRNAs or proteins are still missing), therefore only relative amounts are used in the simulations.
6. Choosing initial values refers to physiological conditions (i.e. S=0). The study of different mutant phenotypes can be done by varying the appropriate parameters (separating the mutants) and using the same equations (for exact values see the codes).

**References**

1. Tyson JJ, Chen K, Novak B. Network dynamics and cell physiology. Nature Rev Mol Cell Biol. 2001;2:908-16.

2. Strogatz SH. Nonlinear Dynamics and Chaos. Reading, MA: Addison-Wesley Co.; 1994.

3. Tyson JJ, Chen KC, Novak B. Sniffers, buzzers, toggles and blinkers: dynamics of regulatory and signaling pathways in the cell. Current Opinion in Cell Biology. 2003;15(2):221-31.

4. Segel IH. Enzyme kinetics behavior and analysis of rapid equilibrium and steady state enzyme systems: Wiley; 1975.

5. Goldbeter A, Koshland DE, Jr. An amplified sensitivity arising from covalent modification in biological systems. Proc Natl Acad Sci U S A. 1981;78(11):6840-4.

6. Tyson JJ, Csikasz-Nagy A, Novak B. The dynamics of cell cycle regulation. BioEssays. 2002;24:1095-109.

7. Ferrell JE, Jr. Tripping the switch fantastic: how a protein kinase cascade can convert graded inputs into switch-like outputs. Trends Biochem Sci. 1996;21(12):460-6.

8. Kapuy O, Barik D, Sananes MR, Tyson JJ, Novak B. Bistability by multiple phosphorylation of regulatory proteins. Prog Biophys Mol Biol. 2009;100(1-3):47-56. Epub 2009 Jun 11.

9. Kaplan D, Glass L. Understanding Nonlinear Dynamics. New York: Springer-Verlag; 1995. Chapter 5 p.

10. Bustin SA, Benes V, Garson JA, Hellemans J, Huggett J, Kubista M, et al. The MIQE guidelines: minimum information for publication of quantitative real-time PCR experiments. Clin Chem. 2009;55(4):611-22.

**II. Mathematical codes for computational simulations**

*The code for simulating time series when GADD34-ATF4 positive and CHOP-ATF4 negative feedback loops are not present*

# a model to simulate ER stress response mechanism with XPP-AUT

# the code for simulating time series when GADD34-ATF4 positive and CHOP-ATF4 negative feedback loops are not present

# initial conditions

eif2aP=0.0099, mATF4=0.6000, ATF4=0.4251, mGadd34=1.0425, Gadd34=0.2818, mCHOP=2156, CHOP=0.2265

# differential equations

# eif2aP represents the active, phosphorylated form of eif2a

eif2aP' = (kaeif2 + kaeif2'*S/(1+S))*(eif2aT - eif2aP) - (kieif2 + kieif2'*Gadd34)*eif2aP

# mATF4 represents ATF4 mRNA in the cell

mATF4' = ksatf + ksatf'*S/(1+S) - kdatf*mATF4

# ATF4 represents the active ATF4 protein in the cell

ATF4' = kssatf'*mATF4*eif2aP - kddatf*ATF4

# mGADD34 represents GADD34 mRNA in the cell

mGadd34' = ksgadd + ksgadd'*ATF4 - kdgadd*mGadd34

# GADD34 represents the active GADD34 protein in the cell

Gadd34' = kssgadd'*mGadd34 + kssgadd"*mGadd34*CHOP + kssgadd’”*mGadd34*eif2aP - kddgadd* Gadd34

# mCHOP represents CHOP mRNA in the cell

mCHOP' = kschop*siCHOP + kschop'*ATF4*siCHOP + kschop"*S/(1+S)*siCHOP - kdchop*mCHOP

# CHOP represents the active CHOP protein in the cell

CHOP' = ksschop'*mCHOP + ksschop”*mCHOP*eif2aP - kddchop*CHOP

# parameters

# simulating low stress: S=5

# simulating high stress: S=15

# simulating high stress combined with guanabenz treatment: S=15, GB=0.1

# simulating high stress combined with siCHOP: S=15, siCHOP=0.3

p S=0, GB=1, siCHOP=1

p kaeif2=0.001, kaeif2'=0.5, kieif2=0.1, kieif2'=0.1, eif2aT=1

p ksatf=3, ksatf'=2, kdatf=5

p kssatf'=0.15, kddatf=0.1

p ksgadd=0.5, ksgadd'=0.05, kdgadd=0.5

p kssgadd'=0.01, kssgadd"=0.25, kssgadd’”=0.01, kddgadd=0.25

p kschop=0.001, kschop'=0.125, kschop"=0.275, kdchop=0.25

p ksschop'=0.1035, ksschop”=0.001, kddchop=0.1

# numerics

@ TOTAL=120, METH=stiff, delay=50

done

*The code for simulating time series when GADD34-ATF4 positive and CHOP-ATF4 negative feedback loops are present*

# a model to simulate ER stress response mechanism with XPP-AUT

# the code for simulating time series when GADD34-ATF4 positive and CHOP-ATF4 negative feedback loops are present

# initial conditions

eif2aP=0.0099, mATF4=0.6000, ATF4=0.4251, mGadd34=1.0425, Gadd34=0.2818, mCHOP=2156, CHOP=0.2265

# differential equations

# eif2aP represents the active, phosphorylated form of eif2a

eif2aP' = (kaeif2 + kaeif2'*S/(1+S))*(eif2aT - eif2aP) - (kieif2 + kieif2'*Gadd34)*eif2aP

# mATF4 represents ATF4 mRNA in the cell

mATF4' = ksatf + ksatf'*S/(1+S) - kdatf*mATF4

# ATF4 represents the active ATF4 protein in the cell

ATF4' = kssatf'*mATF4*eif2aP + kssatf"*mATF4*Gadd34*GB - (kddatf + kddatf'*CHOP)*ATF4

# mGADD34 represents GADD34 mRNA in the cell

mGadd34' = ksgadd + ksgadd'*ATF4 - kdgadd*mGadd34

# GADD34 represents the active GADD34 protein in the cell

Gadd34' = kssgadd'*mGadd34 + kssgadd"*mGadd34*CHOP + kssgadd’”*mGadd34*eif2aP - kddgadd* Gadd34

# mCHOP represents CHOP mRNA in the cell

mCHOP' = kschop*siCHOP + kschop'*ATF4*siCHOP + kschop"*S/(1+S)*siCHOP - kdchop*mCHOP

# CHOP represents the active CHOP protein in the cell

CHOP' = ksschop'*mCHOP + ksschop”*mCHOP*eif2aP - kddchop*CHOP

# parameters

# simulating low stress: S=1

# simulating high stress: S=10

# simulating high stress combined with guanabenz treatment: S=10, GB=0.1

# simulating high stress combined with siCHOP: S=10, siCHOP=0.3

# simulating high stress combined with GADD34-OP: S=10, kssgadd’=0.5

# simulating high stress combined with CHOP-OP: S=10, ksschop’=0.5

p S=0, GB=1, siCHOP=1

p kaeif2=0.001, kaeif2'=0.5, kieif2=0.1, kieif2'=0.1, eif2aT=1

p ksatf=3, ksatf'=2, kdatf=5

p kssatf'=0.05, kssatf"=0.5, kddatf=0.1, kddatf'=0.45

p ksgadd=0.5, ksgadd'=0.05, kdgadd=0.5

p kssgadd'=0.01, kssgadd"=0.25, kssgadd’”=0.01, kddgadd=0.25

p kschop=0.001, kschop'=0.125, kschop"=0.275, kdchop=0.25

p ksschop'=0.1015, ksschop”=0.001, kddchop=0.1

# numerics

@ TOTAL=120, METH=stiff, delay=50

done

*Short description about the parameters of our mathematical models*

| **parameters** | **description** |
| --- | --- |
| **S** | stress level |
| **GB** | the effect of GADD34 inhibition by GB |
| **siCHOP** | the effect of CHOP silencing by siRNA |
| **kaeif2** | background activation of eiF2a-P |
| **kaeif2'** | ER stress dependent activation of eiF2a-P |
| **kieif2** | background inactivation of eiF2a-P |
| **kieif2'** | GADD34-dependent inactivation of eiF2a-P |
| **eif2aT** | total amount of eiF2a |
| **ksatf** | backgorund synthesis of ATF4 mRNA |
| **ksatf'** | ER stress dependent synthesis of ATF4 mRNA |
| **kdatf** | degradation of ATF4 mRNA |
| **kssatf'** | eiF2a-P dependent synthesis of ATF4 protein |
| **kssatf"** | GADD34 dependent synthesis of ATF4 protein |
| **kddatf** | degradation of ATF4 protein |
| **kddatf'** | CHOP dependent degradation of ATF4 protein |
| **ksgadd** | backgorund synthesis of GADD34 mRNA |
| **ksgadd'** | eiF2a-P dependent synthesis of GADD34 mRNA |
| **kdgadd** | degradation of GADD34 mRNA |
| **kssgadd'** | GADD34 mRNA dependent synthesis of GADD34 protein |
| **kssgadd"** | CHOP dependent synthesis of GADD34 protein |
| **kssgadd’”** | eiF2a-P dependent synthesis of GADD34 protein |
| **kddgadd** | degradation of GADD34 protein |
| **kschop** | backgorund synthesis of CHOP mRNA |
| **kschop'** | ATF4 dependent synthesis of CHOP mRNA |
| **kschop"** | ER stress dependent synthesis of CHOP mRNA |
| **kdchop** | degradation of CHOP mRNA |
| **ksschop'** | CHOP mRNA dependent synthesis of CHOP protein |
| **ksschop”** | eiF2a-P dependent synthesis of CHOP protein |
| **kddchop** | degradation of CHOP protein |

**Figures**


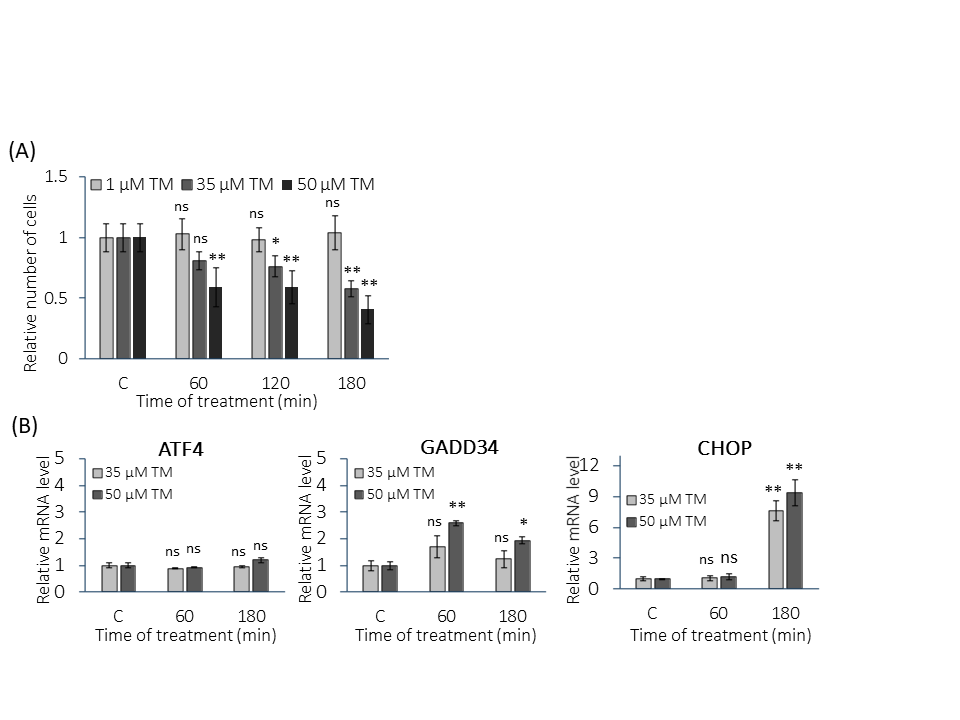


**Figure S1.** **Tunicamycin-induced ER stress decreases cell number and turns on PERK-pathway on mRNA level.** (A) The relative cell number were followed in case of low (light grey column, 1 µM TM for 3 h), medium-high (dark grey column, 35 µM TM for 3 h) and high (black column, 50 µM TM for 3 h) levels of ER stress in HEK293T. (B) The mRNA level of key markers of PERK pathway (i.e. ATF4, CHOP and GADD34) were depicted by real-time PCR in case of medium-high (light grey column, 35 µM TM for 3 h) and high (dark grey column, 50 µM TM for 3 h) level of ER stress in HEK293T for 2 h. For each of the experiments, three complete biological repeat experiments were carried out. Error bars represent standard deviation. Results are presented as mean values ± S.D. and were compared using ANOVA with Tukey’s multiple comparison post hoc test. Asterisks indicate statistically significant difference from the appropriate control: * p < 0.05; ** p < 0.01.

###
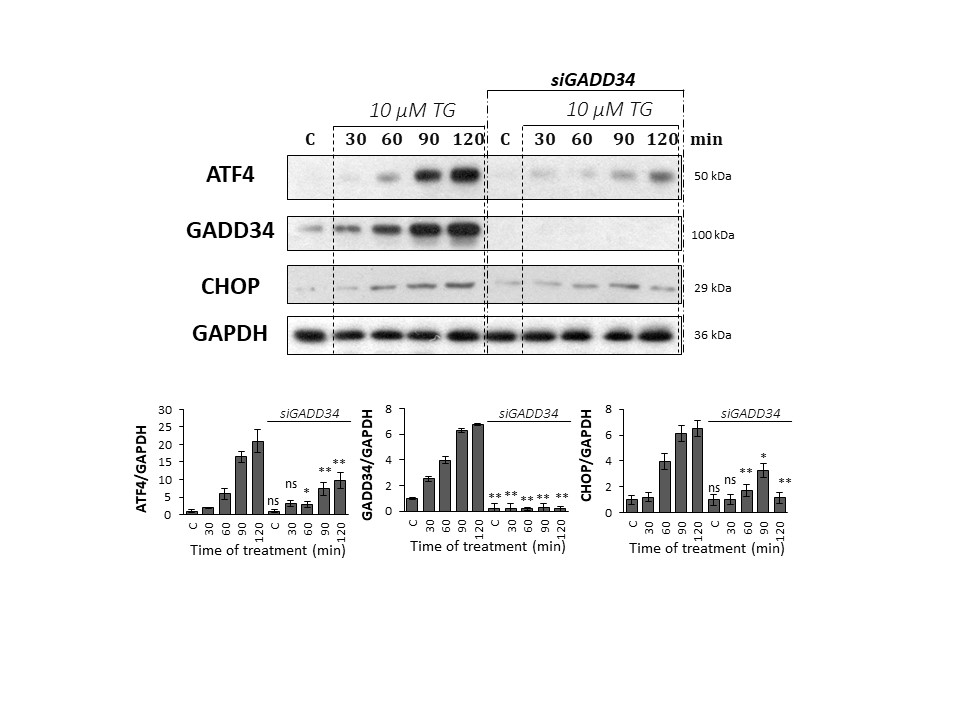


### Figure S2. Excessive level of ER stress (10 µM TG) combined with RNA silencing of GADD34 affects PERK pathway on protein level.

GADD34 silencing via siRNA was combined with high level of ER stress (10 µM TG for 2 h) in HEK293T cells and then the cells were denoted in time. Control samples were not treated with any reagent (including DMSO). **(upper panel)** The markers of PERK pathway (ATF4, CHOP and GADD34) were followed by immunoblotting. GAPDH was used as loading control. The full-length blots are included in a Supplementary Information. **(lower panel)** Densitometry data represents the intensity of ATF4, CHOP and GADD34 normalized for GAPDH. Error bars represent standard deviation. In the calculation of statistical significance, data from GADD34-silenced samples were compared to the non-silenced samples with the corresponding treatment time of 10 µM thapsigargin (eg. GADD34-silenced control was compared to non-silenced control, and thapsigargin-treated samples with the combination of GADD34-silencing were compared to the non-silenced samples treated with thapsigargin for the same period of time). TG is an abbreviation for thapsigargin. For each of the experiments, three complete biological repeat experiments were carried out. Error bars represent standard deviation. Results are presented as mean values ± S.D. and were compared using ANOVA with Tukey’s multiple comparison post hoc test. Asterisks indicate statistically significant difference from the appropriate control: * p < 0.05; ** p < 0.01.

###
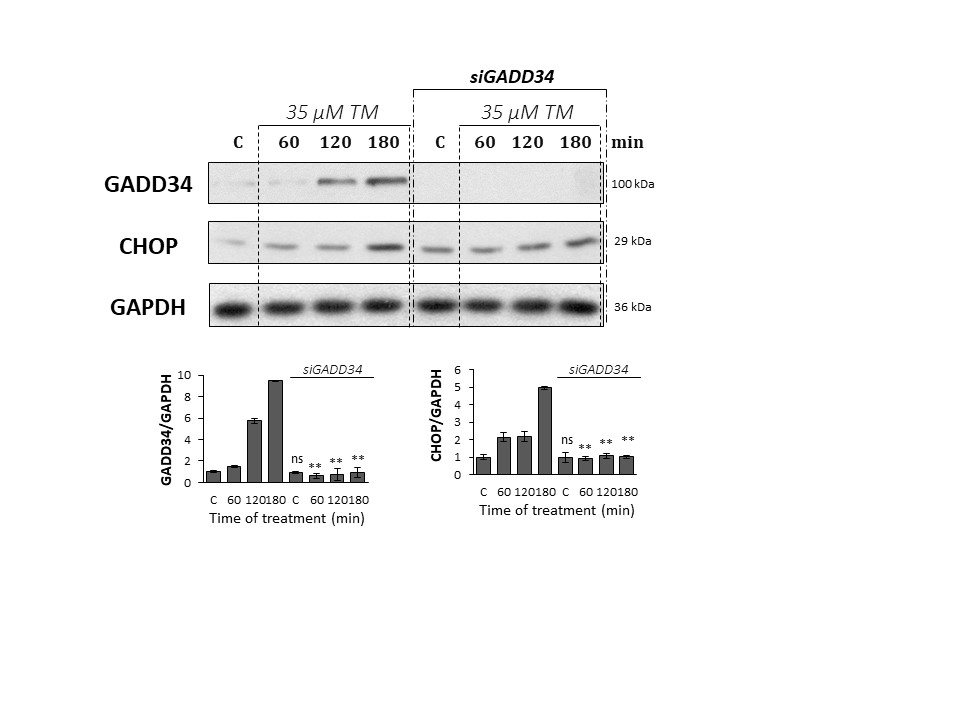


### Figure S3. Excessive level of ER stress (35 µM TM) combined with RNA silencing of GADD34 affects PERK pathway on protein level.

GADD34 silencing via siRNA was combined with high level of ER stress (35 µM TM for 3 h) in HEK293T cells and then the cells were denoted in time. Control samples were not treated with any reagent (including DMSO). **(upper panel)** The markers of PERK pathway (CHOP and GADD34) were followed by immunoblotting. GAPDH was used as loading control. The full-length blots are included in a Supplementary Information. **(lower panel)** Densitometry data represents the intensity of CHOP and GADD34 normalized for GAPDH. Error bars represent standard deviation. In the calculation of statistical significance, data from GADD34-silenced samples were compared to the non-silenced samples with the corresponding treatment time of 10 µM thapsigargin (eg. GADD34-silenced control was compared to non-silenced control, and thapsigargin-treated samples with the combination of GADD34-silencing were compared to the non-silenced samples treated with thapsigargin for the same period of time). TG is an abbreviation for thapsigargin. For each of the experiments, three complete biological repeat experiments were carried out. Error bars represent standard deviation. Results are presented as mean values ± S.D. and were compared using ANOVA with Tukey’s multiple comparison post hoc test. Asterisks indicate statistically significant difference from the appropriate control:
** p < 0.01.


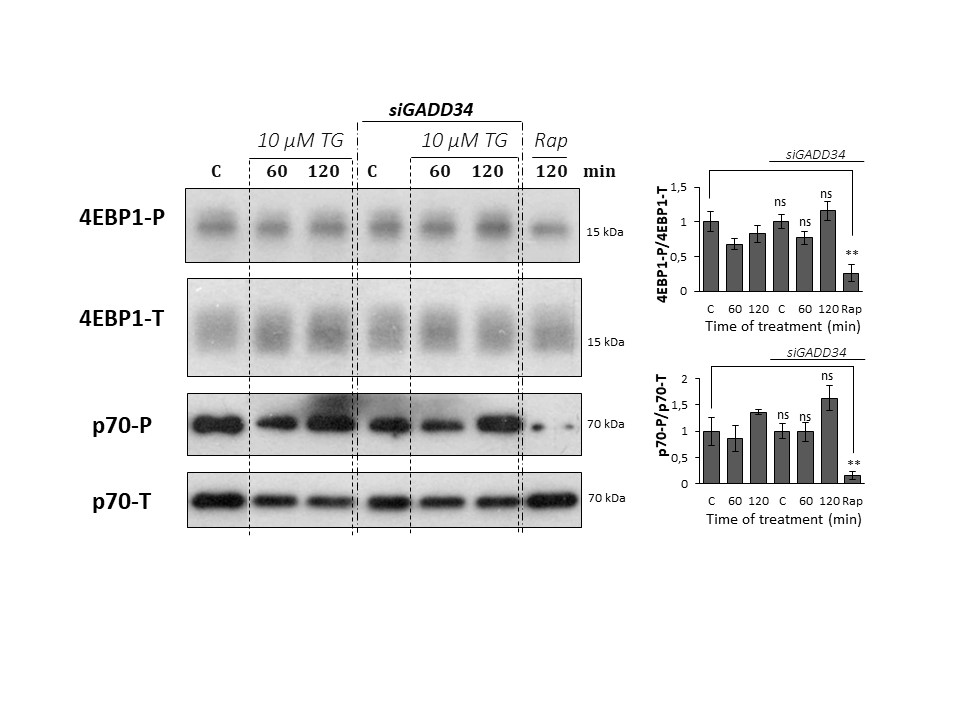


### Figure S4. Excessive level of ER stress (10 µM TG) combined with RNA silencing of GADD34 does not affect mTOR on protein level.

GADD34 silencing via siRNA was combined with high level of ER stress (10 µM TG for 2 h) in HEK293T cells and then the cells were denoted in time. Positive control for mTOR inhibition (Rap) was treated with 100 nM Rapamycin for 120 min. Control samples were not treated with any reagent (including DMSO). **(left panel)** The phosphorylation of mTOR substrates (4EBP1 (Thr37/46) and p70-P) were followed by immunoblotting. The full-length blots are included in a Supplementary Information. **(right panel)** Densitometry data represents the intensity of 4EBP1-P (upper band), and p70 normalized for the total level of 4EBP1 or p70. Error bars represent standard deviation. In the calculation of statistical significance, data from GADD34-silenced samples were compared to the non-silenced samples with the corresponding treatment time of 10 µM thapsigargin (eg. GADD34-silenced control was compared to non-silenced control, and thapsigargin-treated samples with the combination of GADD34-silencing were compared to the non-silenced samples treated with thapsigargin for the same period of time). Data from Rapamycin-treated sample were compared to non-silenced, untreated control. TG is an abbreviation for thapsigargin. For each of the experiments, three complete biological repeat experiments were carried out. Error bars represent standard deviation. Results are presented as mean values ± S.D. and were compared using ANOVA with Tukey’s multiple comparison post hoc test. Asterisks indicate statistically significant difference from the appropriate control:
** p < 0.01.


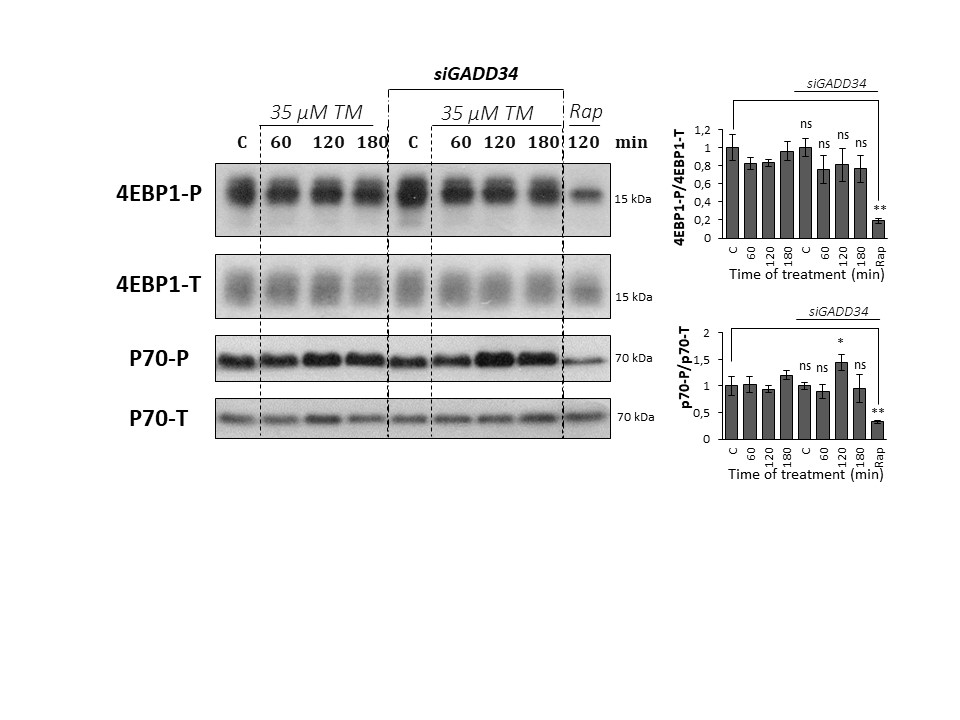


### Figure S5. Excessive level of ER stress (35 µM TM) combined with RNA silencing of GADD34 does not affect mTOR on protein level.

GADD34 silencing via siRNA was combined with high level of ER stress (35 µM TM for 3 h) in HEK293T cells and then the cells were denoted in time. Positive control for mTOR inhibition (Rap) was treated with 100 nM Rapamycin for 120 min. Control samples were not treated with any reagent (including DMSO). **(left panel)** The phosphorylation of mTOR substrates (4EBP1 (Thr37/46) and p70-P) were followed by immunoblotting. The full-length blots are included in a Supplementary Information. **(right panel)** Densitometry data represents the intensity of 4EBP1-P (upper band), and p70 normalized for the total level of 4EBP1 or p70. Error bars represent standard deviation. In the calculation of statistical significance, data from GADD34-silenced samples were compared to the non-silenced samples with the corresponding treatment time of 10 µM thapsigargin (eg. GADD34-silenced control was compared to non-silenced control, and thapsigargin-treated samples with the combination of GADD34-silencing were compared to the non-silenced samples treated with thapsigargin for the same period of time). Data from Rapamycin-treated sample were compared to non-silenced, untreated control. TG is an abbreviation for thapsigargin. For each of the experiments, three complete biological repeat experiments were carried out. Error bars represent standard deviation. Results are presented as mean values ± S.D. and were compared using ANOVA with Tukey’s multiple comparison post hoc test. Asterisks indicate statistically significant difference from the appropriate control:
** p < 0.01.


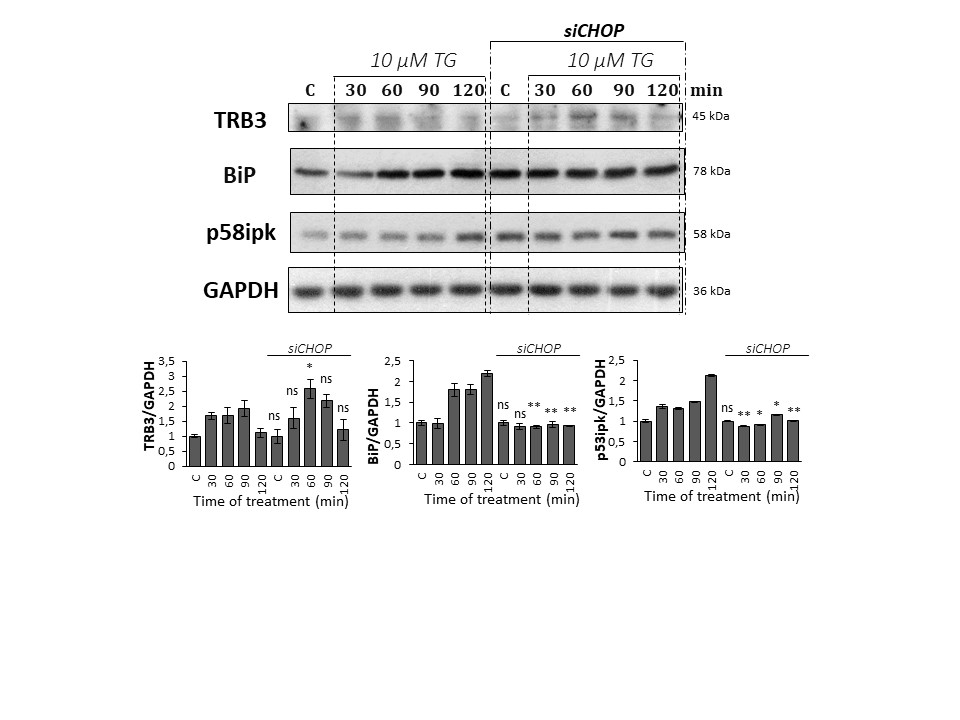


### Figure S6. The effect of CHOP silencing combined with excessive level of ER stress (10 µM TG) on ATF4 substrate TRB3 and ISR non-related ER stress marker BiP and p58ipk.

CHOP silencing via siRNA was combined with high level of ER stress (10 µM TG for 2 h) in HEK293T cells and then the cells were denoted in time. Control samples were not treated with any reagent (including DMSO). **(upper panel)** TRB3 (ATF4 substrate), BiP and p58ipk (ISR non-related ER stress markers) were followed by immunoblotting. GAPDH was used as loading control. The full-length blots are included in a Supplementary Information. **(lower panel)** Densitometry data represents the intensity of TRB3, BiP and p58ipk normalized for GAPDH. Error bars represent standard deviation. In the calculation of statistical significance, data from CHOP-silenced samples were compared to the non-silenced samples with the corresponding treatment time of 10 µM thapsigargin (eg. CHOP-silenced control was compared to non-silenced control, and thapsigargin-treated samples with the combination of CHOP-silencing were compared to the non-silenced samples treated with thapsigargin for the same period of time). TG is an abbreviation for thapsigargin. For each of the experiments, three complete biological repeat experiments were carried out. Error bars represent standard deviation. Results are presented as mean values ± S.D. and were compared using ANOVA with Tukey’s multiple comparison post hoc test. Asterisks indicate statistically significant difference from the appropriate control: * p < 0.05; ** p < 0.01.


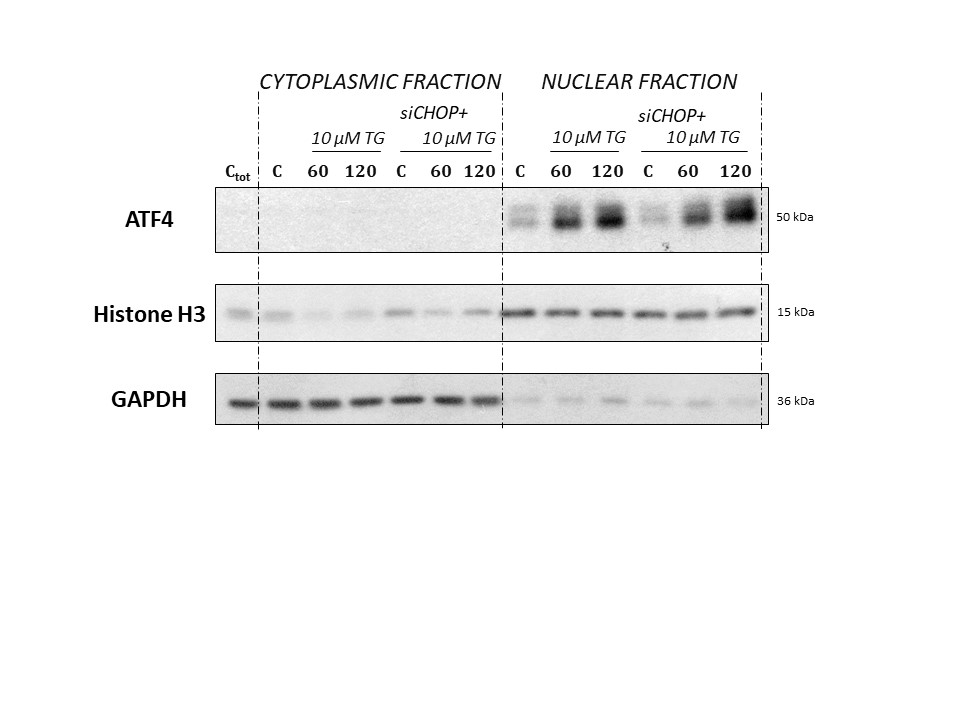


### Figure S7. CHOP silencing does not affect on the translocation of ATF4 to the nucleus during excessive level of ER stress (10 µM TG).

CHOP silencing via siRNA was combined with high level of ER stress (10 µM TG for 2 h) in HEK293T cells and then the cells were denoted in time. Control samples were not treated with any reagent (including DMSO). Protein was isolated separately from the cytoplasm and nucleus of the cells (as cytoplasmic and nuclear fraction). Ct means total protein extract from a control sample. ATF4, Histone H3 and GAPDH were followed by immunoblotting. GAPDH was used as control protein of cytoplasmic fraction and Histone H3 was used as a control protein of nuclear fraction, for the verification of the efficiency of the separation. The full-length blots are included in a Supplementary Information.


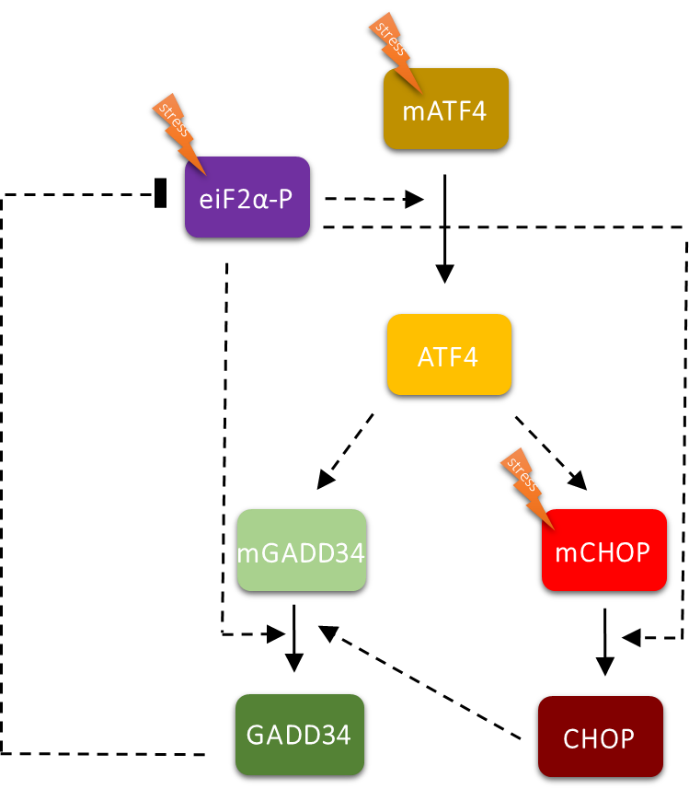


**Figure S8. The wiring diagram of regulatory network of PERK pathway controlled ER stress response mechanism when GADD34-ATF4 positive and CHOP-ATF4 negative feedback loops are not present.** The regulatory elements are denoted by isolated coloured boxes. Dashed line shows how the components can influence each other, while blocked end lines denote inhibition.


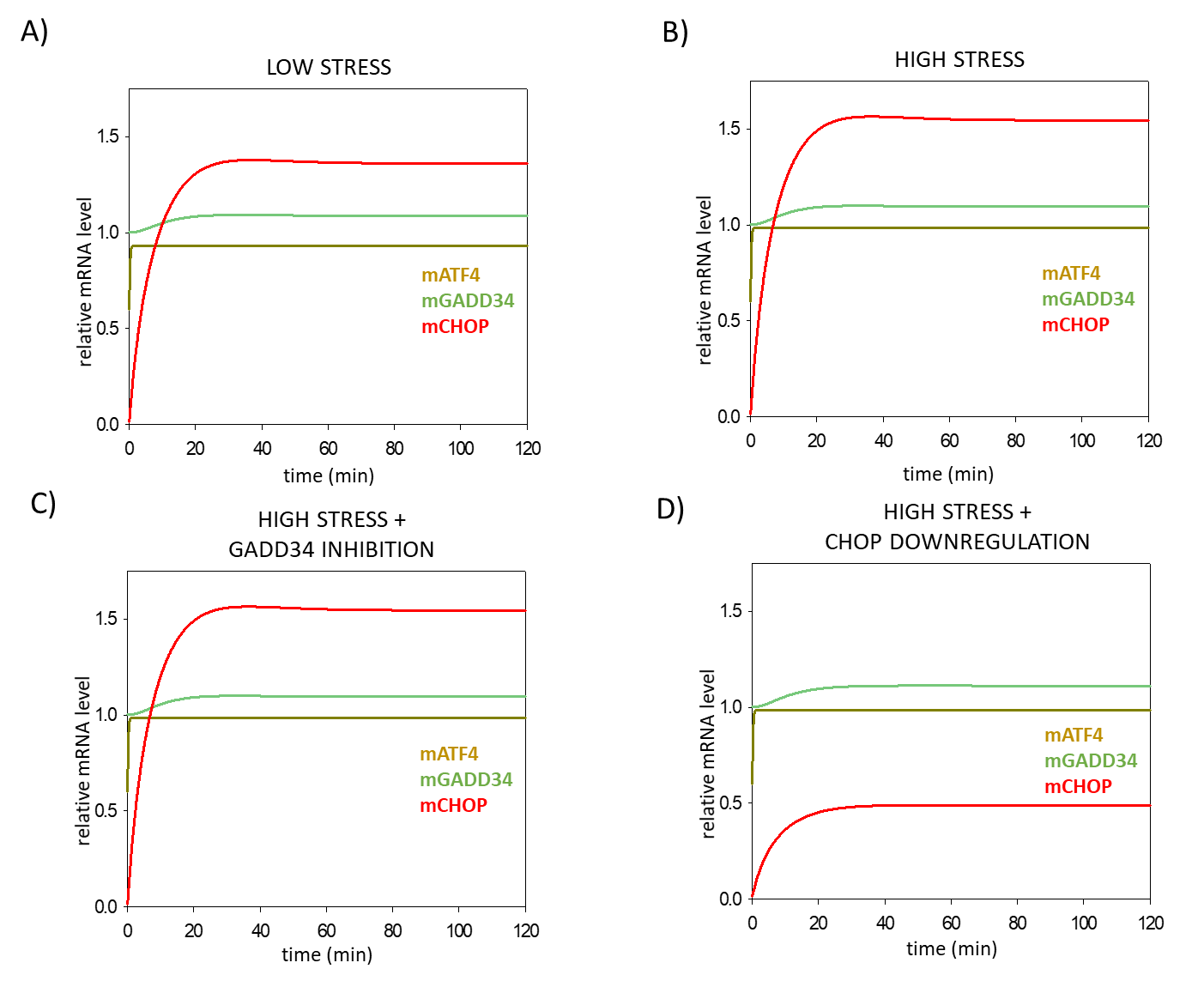


**Figure S9. Computational analysis of regulatory network of PERK pathway controlled ER stress response mechanism when GADD34-ATF4 positive and CHOP-ATF4 negative feedback loops are not present.** Numerical simulations of **(A-D)** mRNA levels (i.e. mRNA of ATF4, CHOP and GADD34) during various ER stress events (about parameter values see the code found in Supplementary Information). **(A)** represents tolerable level of ER, while **(B)** refers to excessive level of ER stress. High level of ER stress was also combined **(C)** with guanabenz treatment or **(D)** CHOP silencing.

**
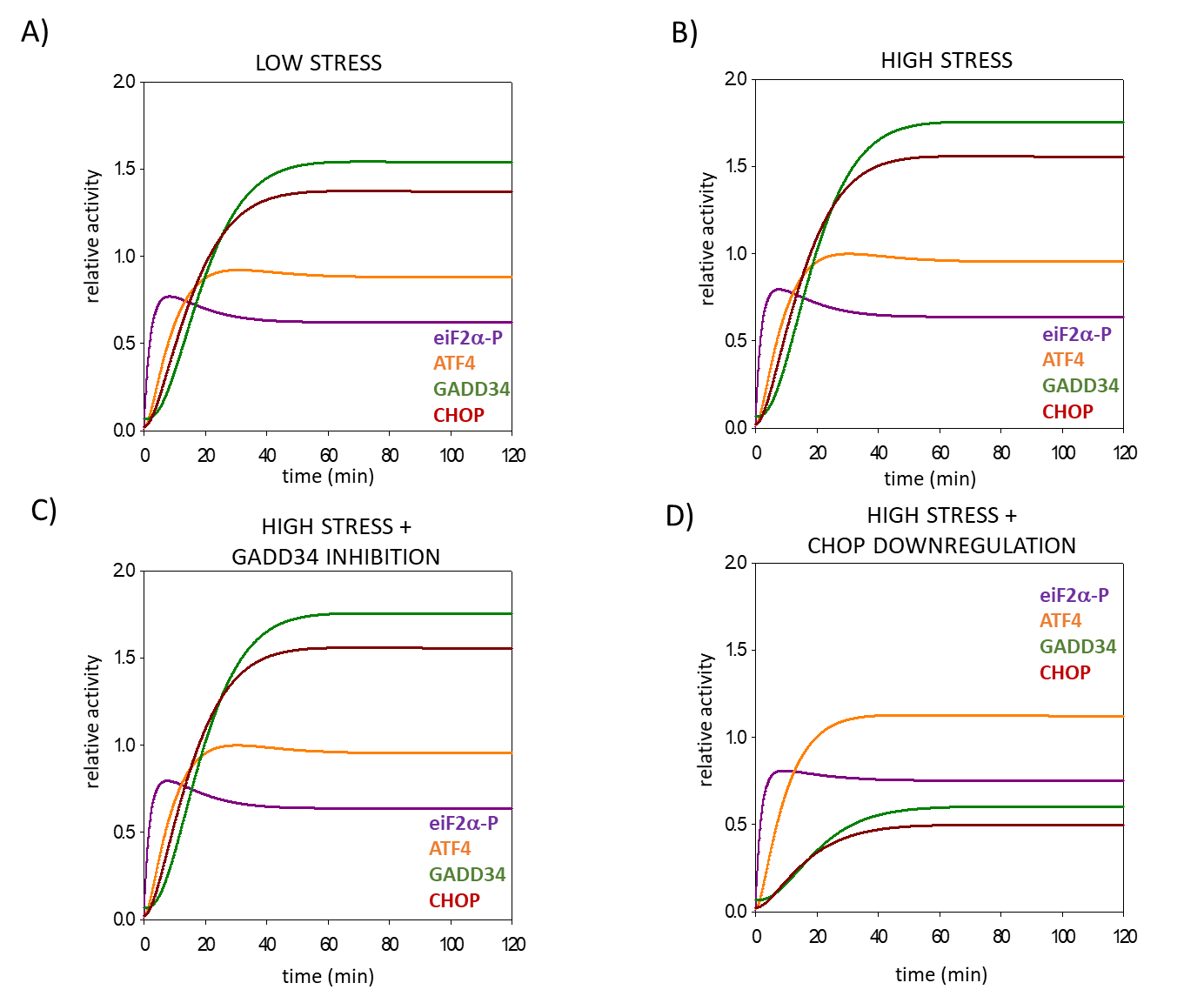
**

**Figure S10. Computational analysis of regulatory network of PERK pathway controlled ER stress response mechanism when GADD34-ATF4 positive and CHOP-ATF4 negative feedback loops are not present.** Numerical simulations of **(A-D)** protein activities (such as eiF2α-P, ATF4, CHOP and GADD34) during various ER stress events (about parameter values see the code found in Supplementary Information). **(A)** represents tolerable level of ER, while **(B)** refers to excessive level of ER stress. High level of ER stress was also combined **(C)** with guanabenz treatment or **(D)** CHOP silencing.

**
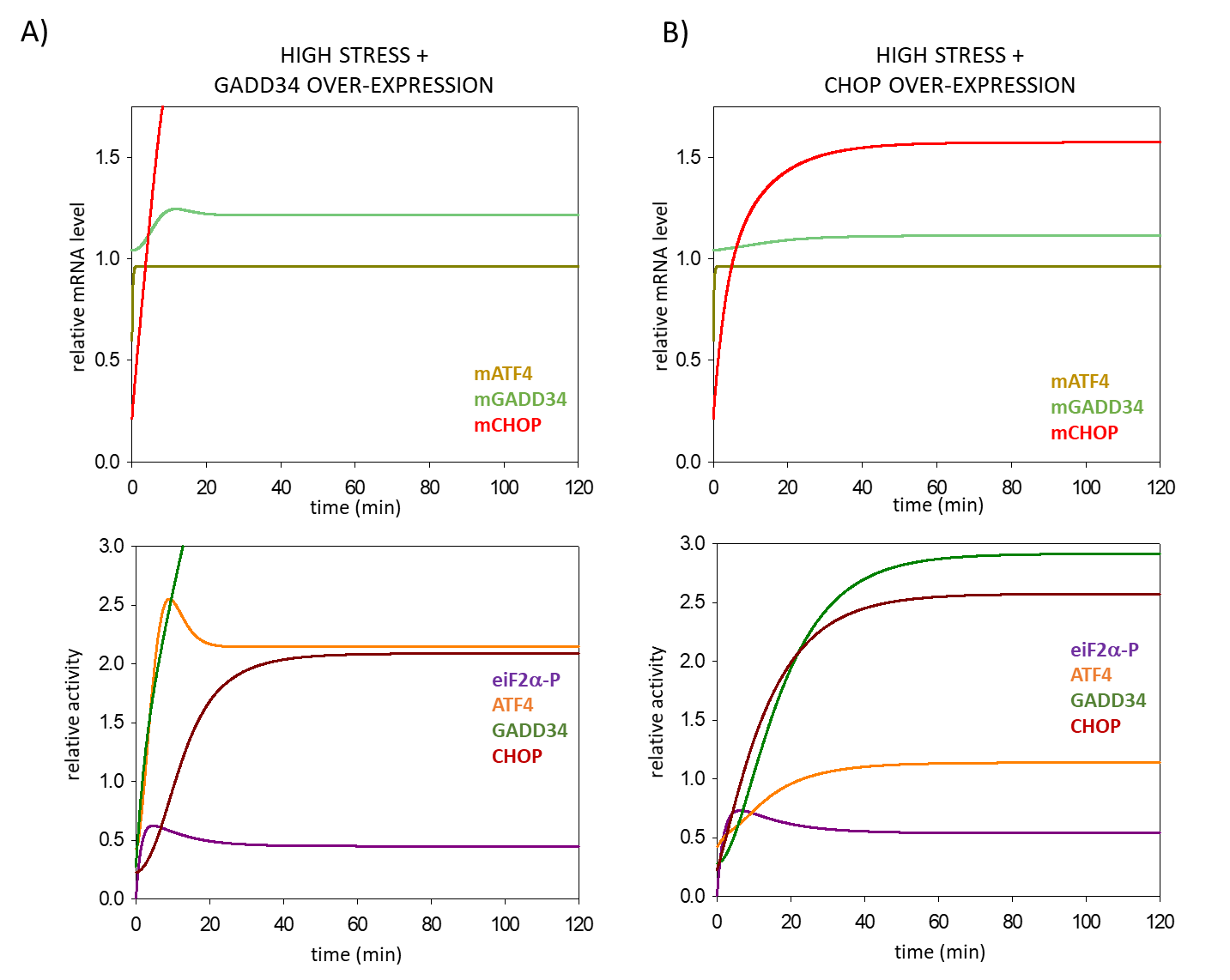
**

**Figure S11. Computational analysis of regulatory network of PERK pathway controlled ER stress response mechanism when GADD34-ATF4 positive and CHOP-ATF4 negative feedback loops are present.** Numerical simulations of **(A-B, upper panel)** mRNA levels (i.e. mRNA of ATF4, CHOP and GADD34) and **(A-B, lower panel)** protein activities (such as eiF2α-P, ATF4, CHOP and GADD34) during various ER stress events (about parameter values see the code found in Supplementary Information). **(A)** represents high level of ER stress combined with GADD34-OP, while **(B)** refers to excessive level of ER stress combined with CHOP over-expression.

**Tables**

**Proteins changing the most in response to 2 hour long 10 μM Thapsigargin treatment in the GADD34-TurboID proximity-labeling experiment in HEK293T cells.**

| **Acc #** | **Protein name** |
| --- | --- |
| **P30153** | Serine/threonine-protein phosphatase 2A 65 kDa regulatory subunit A alpha isoform |
| **Q9Y262** | Eukaryotic translation initiation factor 3 subunit L |
| **P18669** | Phosphoglycerate mutase 1 |
| **P00367** | Glutamate dehydrogenase 1, mitochondrial |
| **P54819** | Adenylate kinase 2, mitochondrial |
| **Q99497** | Protein/nucleic acid deglycase DJ-1 |
| **P40939** | Trifunctional enzyme subunit alpha, mitochondrial |
| **P11177** | Pyruvate dehydrogenase E1 component subunit beta, mitochondrial |
| **P49755** | Transmembrane emp24 domain-containing protein 10 |
| **Q15750** | TGF-beta-activated kinase 1 and MAP3K7-binding protein 1 |
| **P10321** | HLA class I histocompatibility antigen, Cw-7 alpha chain |
| **O00299** | Chloride intracellular channel protein 1 |
| **Q9GZR7** | ATP-dependent RNA helicase DDX24 |
| **P36404** | ADP-ribosylation factor-like protein 2 |
| **O15511** | Actin-related protein 2/3 complex subunit 5 |
| **Q96GA3** | Protein LTV1 homolog |
| **Q05D08** | PA2G4 protein (Fragment) |
| **O75663** | TIP41-like protein |
| **Q15369** | Elongin-C |
| **O00505** | Importin subunit alpha-4 |
| **Q96A33** | Coiled-coil domain-containing protein 47 |
| **P68400** | Casein kinase II subunit alpha |
| **Q9UI30** | Multifunctional methyltransferase subunit TRM112-like protein |
| **P04049** | RAF proto-oncogene serine/threonine-protein kinase |
| **P60468** | Protein transport protein Sec61 subunit beta |

**Table S1. The first 25 proteins showing decrease in their proximity-dependent biotinylation in response to thapsigargin treatment.** Our results suggest that these proteins might decrease their interaction level with GADD34 due to high ER stress.

| **Acc #** | **Protein name** |
| --- | --- |
| **Q01469** | Fatty acid-binding protein 5 |
| **Q08188** | Protein-glutamine gamma-glutamyltransferase E |
| **P04040** | Catalase |
| **Q6UWP8** | Suprabasin |
| **O60573** | Eukaryotic translation initiation factor 4E type 2 |
| **Q05BK6** | TFG protein |
| **Q6E0U4** | Dermokine |
| **Q8TC04** | Keratin 23 (Histone deacetylase inducible) |
| **O75190** | DnaJ homolog subfamily B member 6 |
| **Q9C0F1** | Centrosomal protein of 44 kDa |
| **Q9UJ70** | N-acetyl-D-glucosamine kinase |
| **P31944** | Caspase-14 |
| **O60256** | Phosphoribosyl pyrophosphate synthase-associated protein 2 |
| **P53597** | Succinate--CoA ligase [ADP/GDP-forming] subunit alpha, mitochondrial |
| **Q6ZVX7** | F-box only protein 50 |
| **Q53EV6** | Basic leucine zipper nuclear factor 1 variant (Fragment) |
| **Q9NR12** | PDZ and LIM domain protein 7 |
| **Q9H0W8** | Protein SMG9 |
| **P54646** | 5'-AMP-activated protein kinase catalytic subunit alpha-2 |
| **Q9NRY4** | Rho GTPase-activating protein 35 |
| **Q8WVV4** | Protein POF1B |
| **P41240** | Tyrosine-protein kinase CSK |
| **Q2TAZ0** | Autophagy-related protein 2 homolog A |
| **O43395** | U4/U6 small nuclear ribonucleoprotein Prp3 |
| **B2RA70** | Tyrosine-protein kinase |

**Table S2. The first 25 proteins showing increase in their proximity-dependent biotinylation in response to thapsigargin treatment.** Our results suggest that these proteins might increase their interaction level with GADD34 due to high ER stress.

**III. Original blots of figures**

**
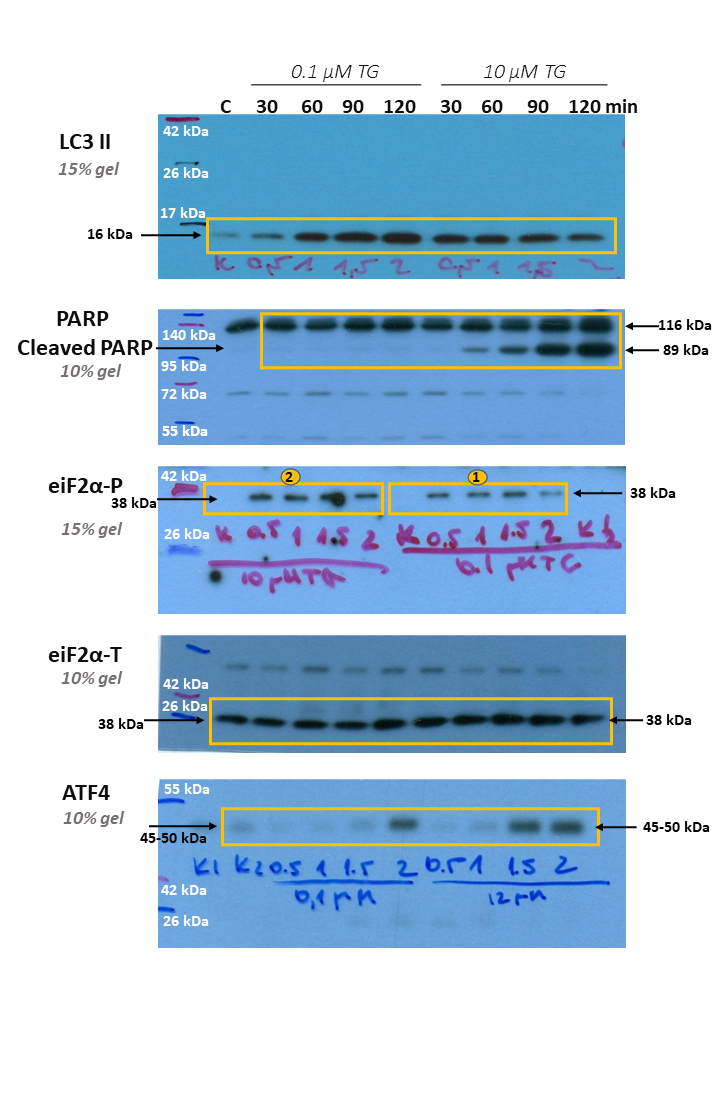
**

**Original version of western blots on Figure 2A** HEK293T cells were treated with low (0.1 µM TG for 2 h) and high (10 µM TG for 2 h) level of ER stress and then cells were denoted in time. LC3, Cleaved PARP, P-eiF2α, T-eiF2α, and ATF4 were followed by immunoblotting. ProSieve Quadcolor Protein Marker (00193837) was used as a protein ladder.

**
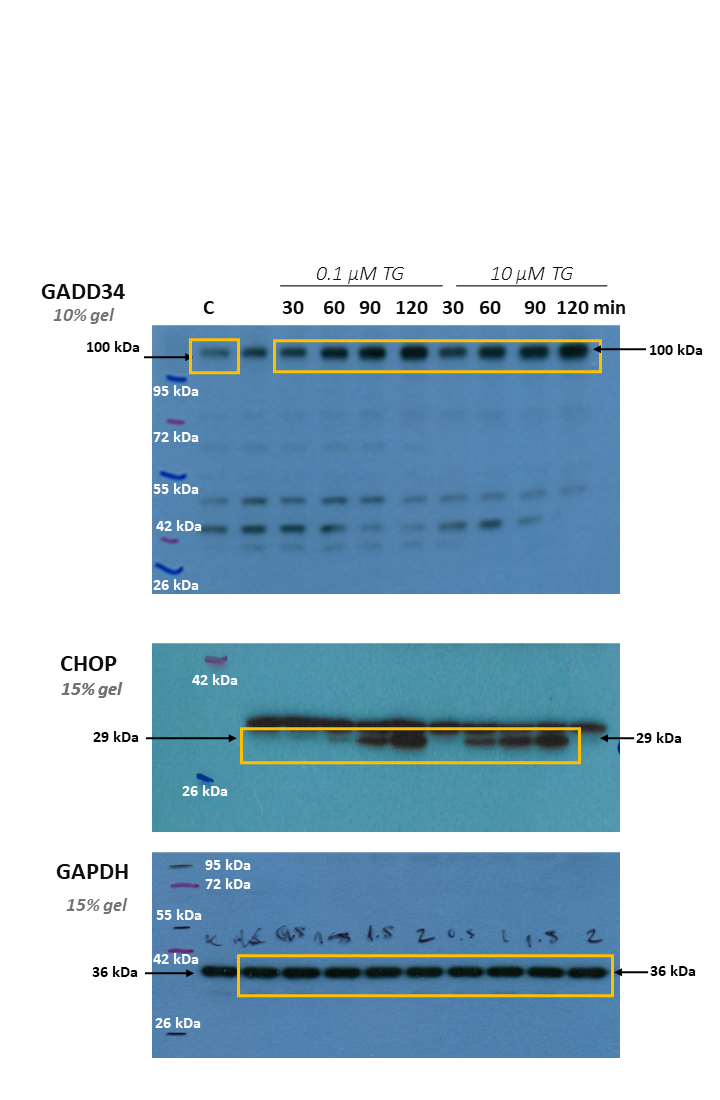
**

**Original version of western blots on Figure 2A** HEK293T cells were treated with low (0.1 µM TG for 2 h) and high (10 µM TG for 2 h) level of ER stress and then cells were denoted in time. GADD34, CHOP, and GAPDH were followed by immunoblotting. ProSieve Quadcolor Protein Marker (00193837) was used as a protein ladder.


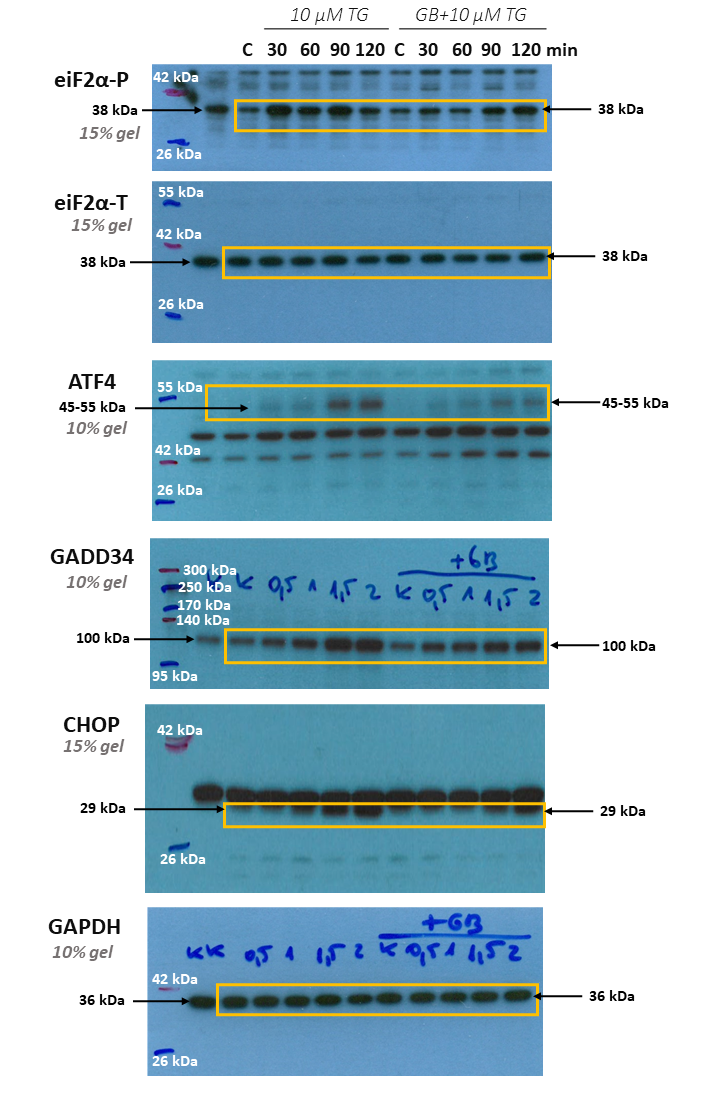


**Original version of western blots on Figure 4A** GADD34 inactivation via guanabenz treatment (50 µM for 1 h) was combined with high level of ER stress (10 µM TG for 2 h) in HEK293T cells and then cells were denoted in time. P-eiF2α, T-eiF2α, ATF4, GADD34, CHOP, and GAPDH were followed by immunoblotting. ProSieve Quadcolor Protein Marker (00193837) was used as a protein ladder.


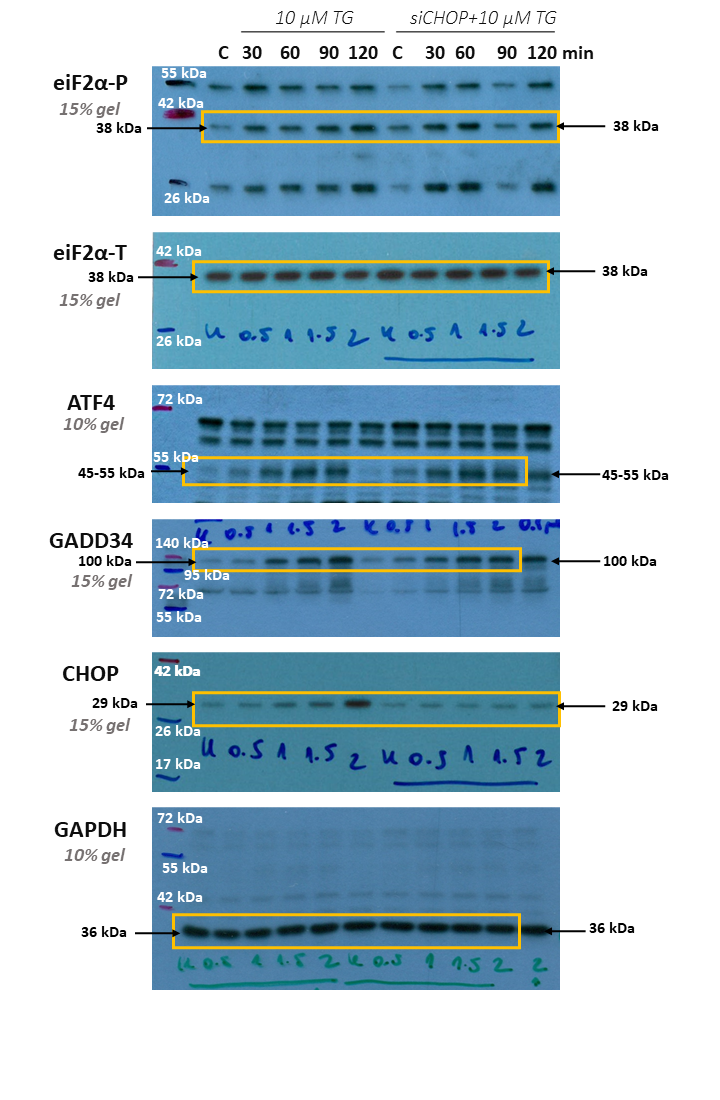


**Original version of western blots on Figure 6A** CHOP was silenced then high level of ER stress (10 µM TG for 2 h) was induced in HEK293T cells and then cells were denoted in time. P-eiF2α, T-eiF2α, ATF4, GADD34, CHOP, and GAPDH were followed by immunoblotting. ProSieve Quadcolor Protein Marker (00193837) was used as a protein ladder.


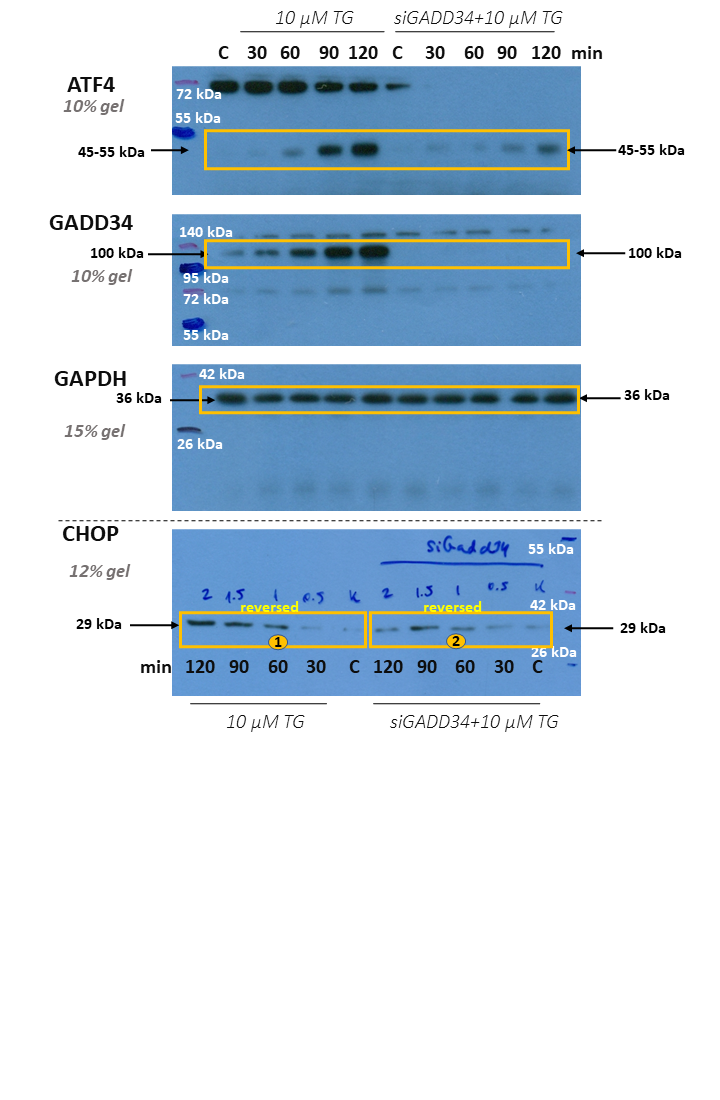


**Original version of western blots on Figure S2** GADD34 silencing via siRNA was combined with high level of ER stress (10 µM TG for 2 h) in HEK293T cells and then the cells were denoted in time. ATF4, CHOP, GADD34 and GAPDH were followed by immunoblotting. ProSieve Quadcolor Protein Marker (00193837) was used as a protein ladder.


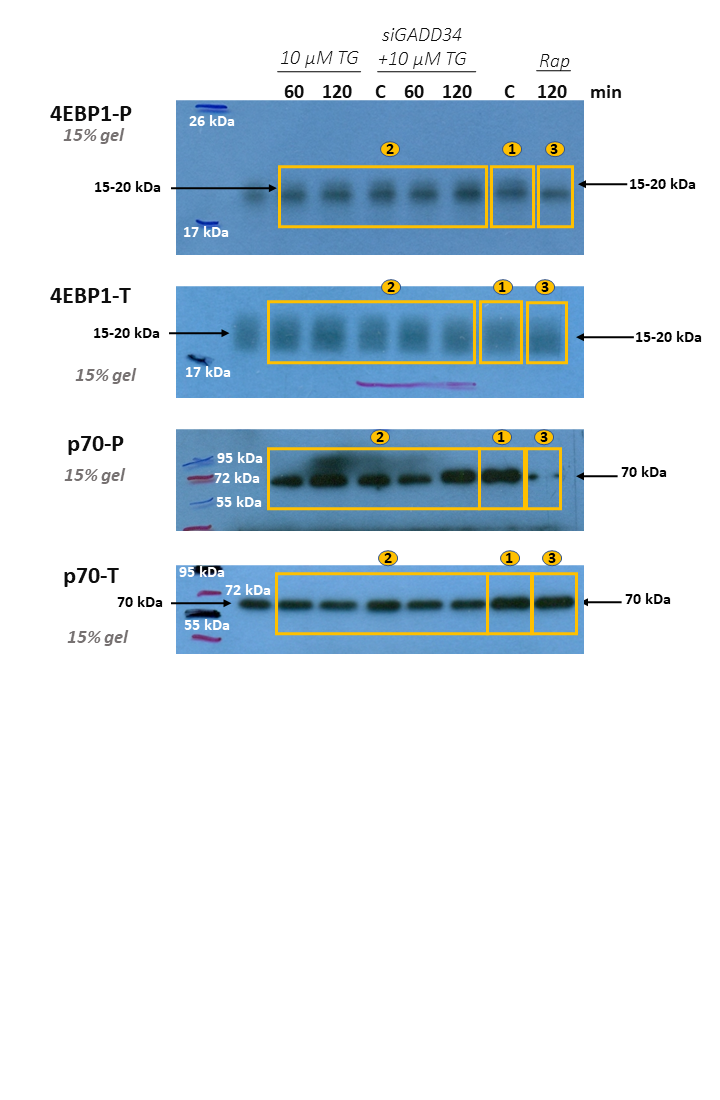


**Original version of western blots on Figure S4** GADD34 silencing via siRNA was combined with high level of ER stress (10 µM TG for 2 h) in HEK293T cells and then the cells were denoted in time. Positive control for mTOR inhibition (Rap) was treated with 100 nM Rapamycin for 120 min. 4EBP1-P, 4EBP1-T, p70-P and p70-T were followed by immunoblotting. ProSieve Quadcolor Protein Marker (00193837) was used as a protein ladder.


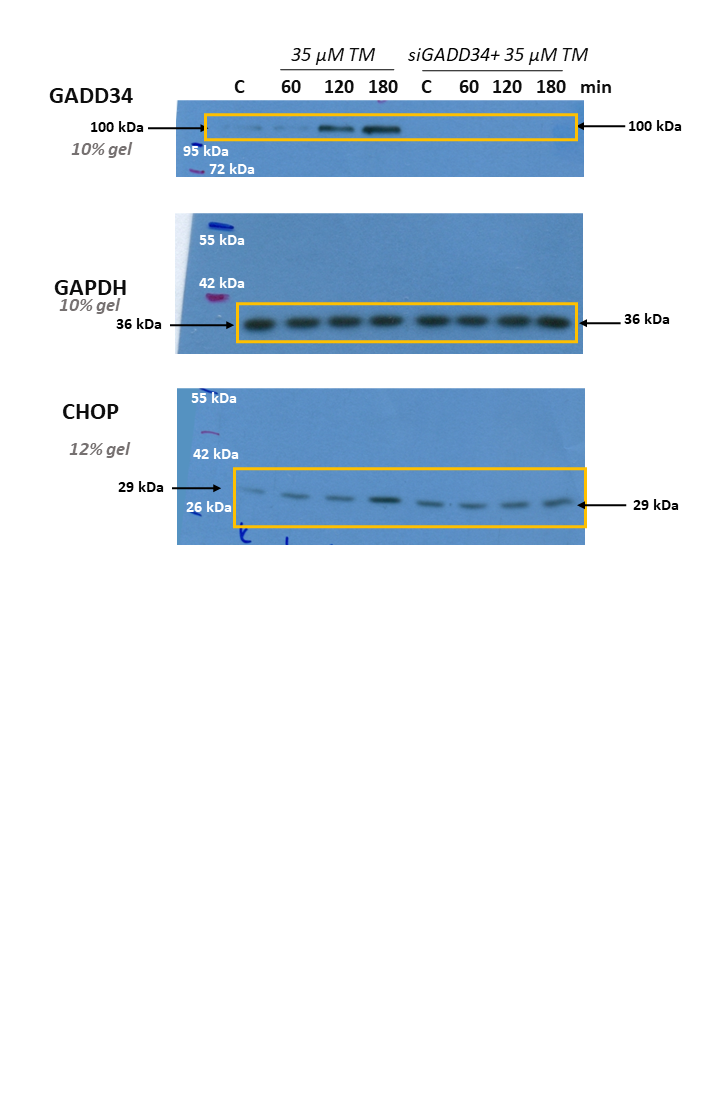


**Original version of western blots on Figure S3** GADD34 silencing via siRNA was combined with high level of ER stress (35 µM TG for 3 h) in HEK293T cells and then the cells were denoted in time. GADD34, GAPDH and CHOP were followed by immunoblotting. ProSieve Quadcolor Protein Marker (00193837) was used as a protein ladder.


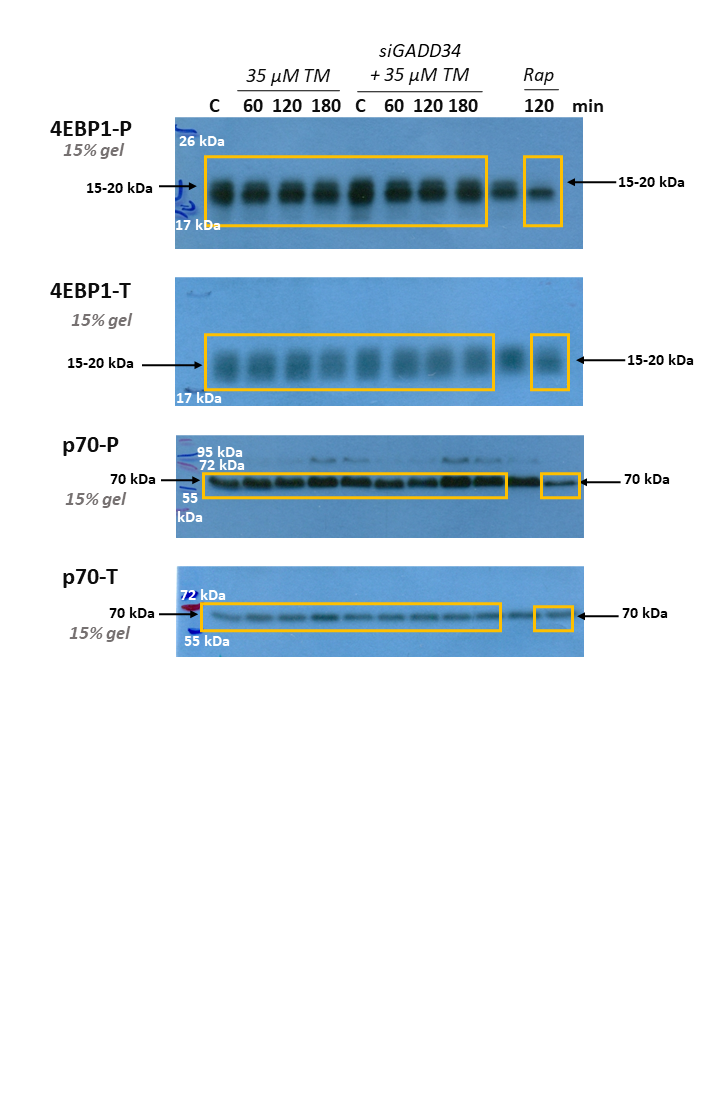


**Original version of western blots on Figure S5** GADD34 silencing via siRNA was combined with high level of ER stress (35 µM TG for 3 h) in HEK293T cells and then the cells were denoted in time. Positive control for mTOR inhibition (Rap) was treated with 100 nM Rapamycin for 120 min. 4EBP1-P, 4EBP1-T, p70-P and p70-T were followed by immunoblotting. ProSieve Quadcolor Protein Marker (00193837) was used as a protein ladder.


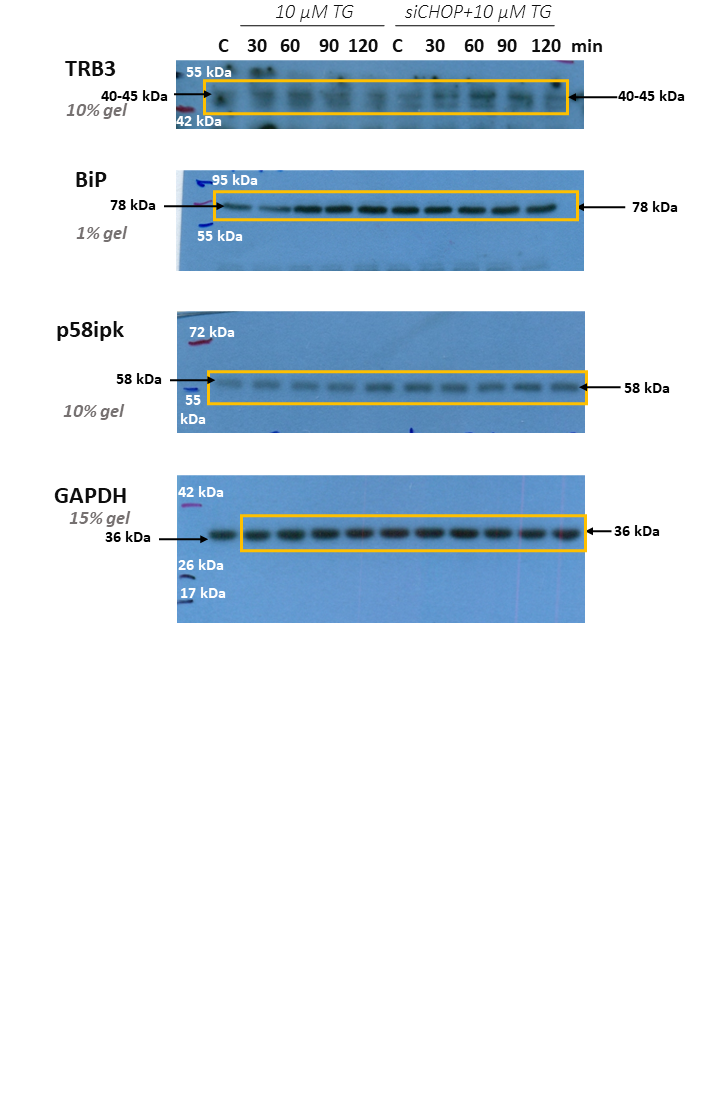


**Original version of western blots on Figure S6** CHOP was silenced then high level of ER stress (10 µM TG for 2 h) was induced in HEK293T cells and then cells were denoted in time. TRB3, BiP, p58ipk and GAPDH were followed by immunoblotting. ProSieve Quadcolor Protein Marker (00193837) was used as a protein ladder.


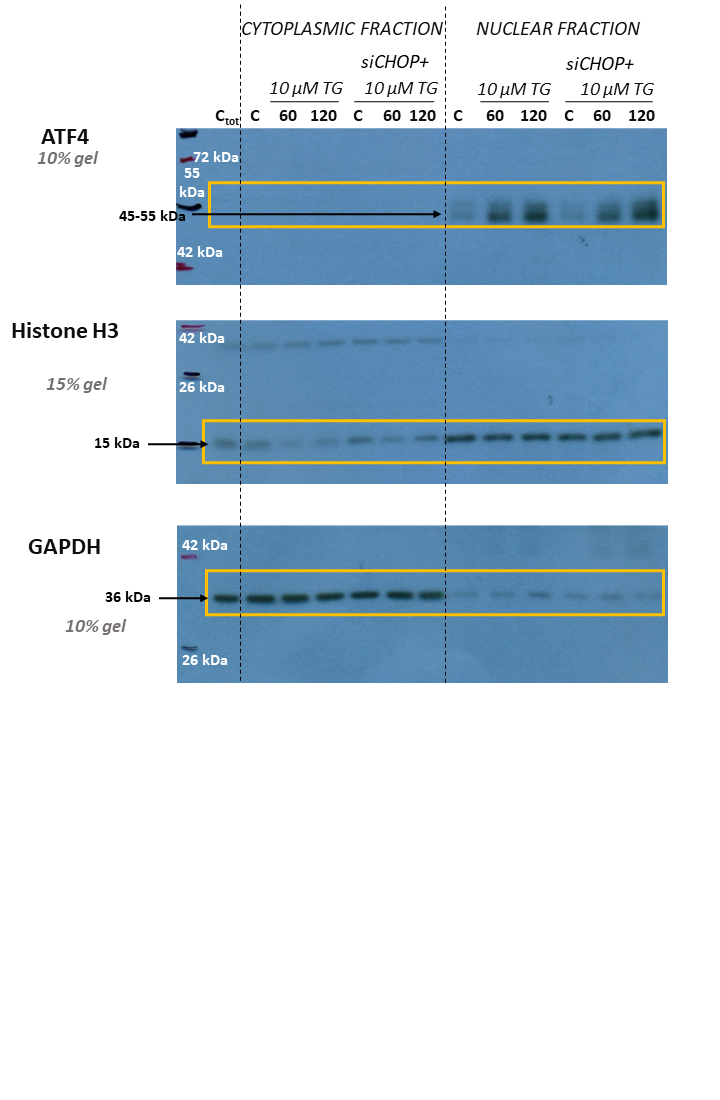


**Original version of western blots on Figure S7** CHOP was silenced then high level of ER stress (10 µM TG for 2 h) was induced in HEK293T cells and then cells were denoted in time. Protein was isolated separately from the cytoplasm and nucleus of the cells (as cytoplasmic and nuclear fraction). Ct means total protein extract from a control sample. ATF4, Histone H3 and GAPDH were followed by immunoblotting. ProSieve Quadcolor Protein Marker (00193837) was used as a protein ladder.

**IV. Efficiency of qPCR primers**


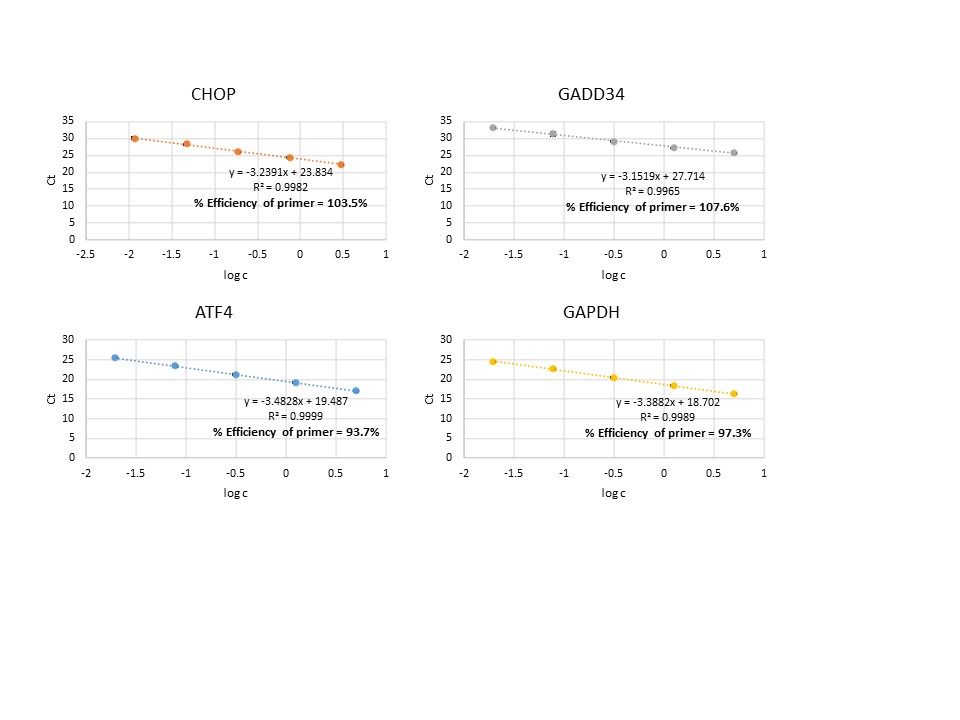


**Quantitative efficiency of qPCR primers.**

For the determination of quantitative efficiency, we performed qPCR in a serial 4x dilutions of cDNA in 5 points for each primer, in three parallel experiments. We used optimized annealing temperature for each primers. The average of Ct values were plotted against the log of concentration of cDNA (log c; ng/reaction). Best fit trend line was generated to the values, and primer efficiency was calculated from the slope of the standard curve. (Pfaffl method)
Efficiency = 10^^(-1/slope)^ – 1, % Efficiency = (E-1)*100 (10)
